# Reassessment of Pioglitazone for Alzheimer’s Disease

**DOI:** 10.3389/fnins.2021.666958

**Published:** 2021-06-16

**Authors:** Ann M. Saunders, Daniel K. Burns, William Kirby Gottschalk

**Affiliations:** ^1^Zinfandel Pharmaceuticals, Inc., Chapel Hill, NC, United States; ^2^Department of Neurology, Duke University School of Medicine, Durham, NC, United States

**Keywords:** Alzheimer’s disease, pioglitazone, preclinical models, observational studies, clinical trials

## Abstract

Alzheimer’s disease is a quintessential ‘unmet medical need’, accounting for ∼65% of progressive cognitive impairment among the elderly, and 700,000 deaths in the United States in 2020. In 2019, the cost of caring for Alzheimer’s sufferers was $244B, not including the emotional and physical toll on caregivers. In spite of this dismal reality, no treatments are available that reduce the risk of developing AD or that offer prolonged mitiagation of its most devestating symptoms. This review summarizes key aspects of the biology and genetics of Alzheimer’s disease, and we describe how pioglitazone improves many of the patholophysiological determinants of AD. We also summarize the results of pre-clinical experiments, longitudinal observational studies, and clinical trials. The results of animal testing suggest that pioglitazone can be corrective as well as protective, and that its efficacy is enhanced in a time- and dose-dependent manner, but the dose-effect relations are not monotonic or sigmoid. Longitudinal cohort studies suggests that it delays the onset of dementia in individuals with pre-existing type 2 diabetes mellitus, which small scale, unblinded pilot studies seem to confirm. However, the results of placebo-controlled, blinded clinical trials have not borne this out, and we discuss possible explanations for these discrepancies.

## Alzheimer’s Disease and Defining the Need

Alzheimer’s disease is a progressive, irreversible neurodegenerative disease whose most fearsome clinical manifestation, and the target of most treatment-oriented human clinical trials, is dementia. Dementia does not respect ethnicities or socioeconomic groups, and the fear of descending into mindlessness is a haunting prospect.

Alzheimer’s disease is, by any definition, an unmet medical need. It is the most common cause of dementia and currently is the third leading cause of death, behind cancer and heart disease. Between 2000 and 2018, the number of deaths attributable to Alzheimer’s disease increased by more than 145%, while the number of deaths attributable to heart disease declined by nearly 8% (Longhe, 2020). In 2018, an excess of 33 million people worldwide lived with AD. Without the development of preventative treatments, this number will soar to 132 million people globally by 2050 (Patterson, 2018). The global annual burden of caring for patients is ∼ 1 trillion USD currently, and is forecast to double by 2030 (Patterson, 2018).

Given this reality, even a small change in the pathophysiological trajectory of an individual with AD would substantially affect both the individual and society. A 1-year delay in the onset of AD could reduce the economic impact in 2030 by $113 billion. By 2050, that 1-year delay would save $219 billion, and 3- and 5-year delays would result in savings of $415 billion and $599 billion, respectively (Zissimopoulos et al., 2015). Because of the high failure rate of treatment studies over the past decade, and in line with the FDA guidelines, the focus of AD clinical research has shifted to early intervention, during the asymptomatic phase of Alzheimer’s disease, rather than initiating treatments after symptoms have emerged (Sperling et al., 2014).

## Challenges to Drug Discovery for Alzheimer’s Disease

Alzheimer’s disease is a heterogeneous disorder that develops over an extended preclinical phase (Sperling et al., 2011; Beason-Held et al., 2013; Thambisetty et al., 2013; Neff et al., 2021). An ‘early onset’ form (EOAD) typically appears before the age of ∼65 years, and is associated with more severe clinical manifestations than the ‘late-onset’ form typically associated with aging. Roughly half of the early onset cases are due to dominantly inherited mutations in any of three genes, presenilin 1 (*PSEN1*), presenilin 2 (*PSEN2*), or amyloid precursor protein (*APP*). *PSEN1* and *PSEN2* modulate the activity of γ-secretase, which processes *APP*. Defects in all three genes result in the accumulation of extracellular deposits of β-amyloid peptides, which are proteolytic products of APP.

Late-onset (or sporadic) Alzheimer’s disease (LOAD) usually appears after the age of 65, and is not associated with the dominant inheritance of any single gene. A number of risk factors, including several genetic risk factors, predispose for Alzheimer’s disease. The most important genetic risk factor is allelic variation in the apolipoprotein E (*APOE*) gene, followed by the rs75932628 (R47H) variant in the Triggering Receptor Expressed in Myeloid cell 2 (*TREM2)* gene. Trem2 is a myeloid cell receptor that binds both ApoE and β-amyloid peptides, and regulates microglial activation. A number of non-genetic determinants also predispose for Alzheimer’s disease, including lack of early life education, hypertension, smoking, obesity, alcohol consumption, and diabetes (Zhang et al., 2021). Biological sex at birth is a significant risk factor for all cause dementia, and women are at greater risk of developing AD than men. Recent genetics findings, including that *APOE* is a risk factor for EOAD (Genin et al., 2011), belie the underlying similarities between EOAD and Alzheimer’s disease (Jansen et al., 2019; Kunkle et al., 2019; Neuner et al., 2020). In the remainder of this review, we will use the term AD to denote late Alzheimer’s disease, and distinguish between EOAD and LOAD when the situation calls.

Multiple hallmarks characterize AD. In addition to extracellular β-amyloid deposits, which is not detected in all cases (Terry et al., 1999; Monsell et al., 2015; Jack et al., 2019; Sperling et al., 2020), and intracellular neurofibrillary tangles (NFT, insoluble deposits of misfolded, hyperphosphorylated tau), AD is characterized by neuronal oxidative stress (Nunomura et al., 2001), neuroinflammation (Heneka et al., 2015a), cerebral insulin resistance (Talbot et al., 2012), and glucose hypometabolism (Mosconi et al., 2008a), calcium overload (Alzheimer’s Association Calcium Hypothesis Workgroup, 2017), mitochondrial malfunction (Swerdlow, 2018) and redistribution (Flannery and Trushina, 2019), synaptic loss (Price et al., 2001), and brain atrophy (Jack et al., 2018). The extent to which any of these factors contributes to AD risk or to manifestations of disease reflects individual variations in biological flexibility and susceptibility to stressors (Neff et al., 2021).

As of 2019, the failure rate of AD drug trials exceeded 99% (Cummings et al., 2014), including the highly ‘validated’ targets amyloid and BACE. These failures reflect knowledge gaps about processes that promote and sustain AD, and how susceptibility to pathogenic determinants varies among individuals. Pan-omics approaches could stratify patient subpopulations according to their underlying pathologies and/or their responses to specific therapies, and identify potential safety issues regarding particular drugs (Roses, 2008). Cancer already uses ‘precision medicine’ approaches (Begum, 2019), and initiatives are underway for other complex diseases (Loscalzo, 2019; Prasad and Groop, 2019; Aletaha, 2020). O’Bryant et al. used similar tools to identify AD subjects who respond to NASID therapy (O’Bryant et al., 2018), but otherwise this approach is not in wide use by the AD clinical research community. Identification of the molecular basis for the heterogeneous nature of AD (Neff et al., 2021), may provide conceptual impetus for adopting it.

**FIGURE 1:**
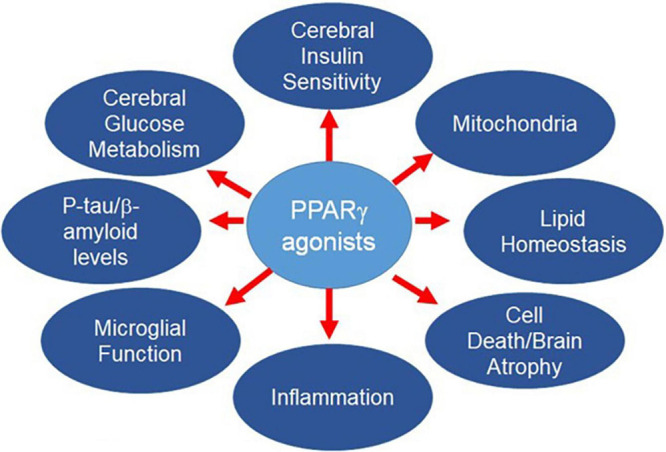
PPARγ regulates many pathways that contribute to AD risk.

The complex pathophysiology of AD means the most successful strategies for lowering AD risk likely will require simultaneous pursuit of multiple targets, as for other multifactorial diseases (American Diabetes Association, 2020; Heidenreich et al., 2020; Unger et al., 2020). However, there are well-known drawbacks to typical polypharmacological approaches, including the appearance of new side effects not seen with the individual drugs, or additive side effects, or diminished efficacy. Adherence to multiple drug regimens may be challenging for prodromal patients and patients with mild or moderate dementia. An alternative approach is to develop a single drug entity that targets multiple disease determinants. PPARγ agonists fulfill this desideratum (Figure 1). After providing an overview of PPAR biology, we will describe AD risk factors and pathophysiological determinants contributing to AD, and the salutary effects of PPARγ agonists. Other agents, such as GLP-1 agonists, also may affect multiple targets in the AD pathogenic pathway. These are outside the scope of the current review, but have been discussed elsewhere (Grieco et al., 2019; Cheng et al., 2020; Yoon et al., 2020).

## Pparγ as a Drug Target for AD

The PPARγ receptor is widely distributed the brain (Braissant et al., 1996; Moreno et al., 2004; Gofflot et al., 2007; Sarruf et al., 2009; Morales-Garcia et al., 2011) and is crucial for learning (He et al., 2009; Jahrling et al., 2014). Activation of the receptor enhances astrocyte/neuron metabolic coupling (Dello Russo et al., 2003; Izawa et al., 2009; Cowley et al., 2012), promotes formation of dendritic spines (Brodbeck et al., 2008), repairs synaptic failure (Chen et al., 2015; Moosecker et al., 2019), corrects LTP impairment (Cowley et al., 2012; Chen et al., 2015), and overcomes the pro-inflammatory, pro-oxidant milieu in the CNS that is central to the pathogenesis of AD. This topic has been reviewed previously (Galimberti and Scarpini, 2017; Cai et al., 2018; Villapol, 2018; Khan et al., 2019).

PPARs constitute a family of three ligand-dependent transcription factors, PPARα, PPARδ and PPARγ, encoded by separate genes and displaying wide, but subtype specific, tissue distribution. PPARs have broad metabolic and anti-inflammatory activities, and are attractive pharmacological targets for treating dyslipidemias (PPARα, Gemfibrozil), type 2 diabetes (PPARγ, pioglitazone, rosiglitazone), and obesity (PPARδ). Pioglitazone and rosiglitazone are high affinity ligands for both PPARγ and PPARα, but are distinguishable in that rosiglitazone is more selective for PPARγ, each agonist regulates bespoke down-stream genes (Verschuren et al., 2014), and pioglitazone enters the brain (Maeshiba et al., 1997; Grommes et al., 2013) to a greater extent than rosiglitazone (Festuccia et al., 2008).

**FIGURE 2:**
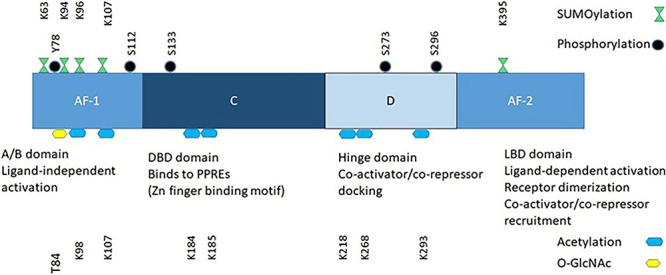
PPARγ covalent modifications.

PPARs recruit and/or enhance the activity of the general transcription machinery of target genes, or repress the expression of others. The PPAR family members share similar structural and mechanistic features (Figure 2). The N-terminal domain contains a ligand-independent transcriptional activation function, AF-1, which is the main determinant of PPAR subtype-selective gene expression. The DNA-binding domain (DBD) binds the receptor to the Peroxisome Proliferator Response Elements (PPRE) of the target genes. It contains the two zinc fingers, which distinguish PPARs from other DNA-binding proteins. PPREs are located either in the gene promoter or in the proximal sequence and contain one or two copies of the consensus sequence 5′-AGAACA-3′. Adjacent to the DBD are the transcriptional cofactor-binding domain (the D site), and the ligand-binding domain (LBD), which mediates binding of the receptor to the PPRE. All three PPARs form obligate heterodimers with RXR receptors. The PPAR and RXR partners bind to the 5′ and 3′ halves of direct repeats of the consensus binding sequence in the PPRE.

PPARγ signaling is non-linear and the net effect depends on fluctuations of PPARγ ligands, on the temporal sequences and durations of post-translational modifications (Figure 2), and on the nature of downstream gene expression networks that interact with the PPARγ transcriptional programs.

## Pparγ and AD-Related Risk Factors

### Introductory Comments

The most significant risk factors for developing Alzheimer’s dementia are potentially non-modifiable and include age, biological sex, a history of AD in first-degree relatives and genetics (Gaugler et al., 2019). The risk for developing AD increases with age (Qiu et al., 2009), and females are at greater risk of developing AD than males (Plassman et al., 2007). Approximately 30 genetic risk loci have been identified (Jansen et al., 2019; Kunkle et al., 2019), which account for only about 65% of the over-all population attributable risk (Livingston et al., 2017). The remainder of the risk is associated with co-morbidities that potentially are modifiable (Livingston et al., 2020). Not surprisingly, there is an underlying connection between biological sex and genetic risk factors for AD. In the first instance, *APOE* ε*4* affects females more severely than men (Farrer et al., 1997; Altmann et al., 2014; Neu et al., 2017). Secondly, recent investigations revealed sex-specific autosomal genetic effects (Zhou et al., 2019; Fan C.C. et al., 2020; Prokopenko et al., 2020). In several instances, risk genes for one sex are not risk genes for the other. For example, the risk haplotype of *PVRL2* was significantly associated with AD in females but not males (Zhou et al., 2019), and *ZBTB7Z*, which encodes a zinc-finger transcription factor, is a risk gene in females but is protective in males (Prokopenko et al., 2020).

### Genetic Risk Factors

The genetic landscape of AD consists of about 30 genomic loci (Lambert et al., 2013; Jansen et al., 2019; Kunkle et al., 2019). PPARγ might be considered a ‘master regulator’ of this genetic landscape because it regulates the expression of at least seven of these genes (Barrera et al., 2018).

#### Early-Onset AD

The histopathological hallmarks of amyloid deposits and NFTs characterize both the ‘Early’ and ‘Late’ onset forms of AD. Causal mutations in three genes, *APP*, *PSEN1* and *PSEN2* contribute to amyloid deposits in the early-onset form (Neuner et al., 2020). *APP* is a cell-surface molecule that is widely distributed throughout the body, and is the precursor molecule of Aβ peptides in the CNS. APP knockout mice do not exhibit a phenotype and its exact role is unknown (O’Brien and Wong, 2011). *PSEN1* and *PSEN2* are catalytic components of the γ-secretase complex, which cooperate with BACE1 (β-site amyloid precursor protein cleaving enzyme) to process amyloid precursor protein and generate the aggregation-prone Aβ peptides found in plaques.

Pioglitazone regulates BACE1–mediated production of Aβ peptides at several levels. The BACE1 gene contains a PPRE and PPARγ controls BACE1 expression. Additionally, CDK5 regulates BACE1 at both the transcriptional and post-transcriptional levels by increasing BACE1 expression (Wen et al., 2008), and regulating β-secretase activity via phosphorylation (Song et al., 2015). Through mechanisms described below, pioglitazone inhibits these CDK5 effects. In both cell-based and *in vivo* models, PPARγ, but not PPARα or PPARδ (Camacho et al., 2004), blocked the generation and release of Aβ peptides (Sastre et al., 2003, 2006; Liu et al., 2013; Gad et al., 2016; Quan et al., 2019b) by blocking BACE1 mRNA and protein expression, and promoting Aβ peptide clearance (Camacho et al., 2004). *In vivo*, PPARγ activation resulted in significantly reduced β-amyloid plaques (Heneka et al., 2005; Escribano et al., 2010; O’Reilly and Lynch, 2012; Searcy et al., 2012; Liu et al., 2013; Quan et al., 2019b). *In vitro*, the RXR ligand *cis*-retinoic acid alone was as effective as PPARγ agonists alone, including pioglitazone (Camacho et al., 2004). In the cell culture, PPARγ agonists blocked increased BACE1 expression and synthesis and release of Aβ peptides elicited by pro-inflammatory cytokines (Sastre et al., 2003). Conversely, PPARγ knock-down increased BACE1 expression (Sastre et al., 2006). Aβ peptides and fibrils are pro-inflammatory and increase CDK5 activation (Quan et al., 2019a), and astrogliosis, microglial damage and neuronal apoptosis (Sastre et al., 2003). Pioglitazone down-regulated CDK5 expression and PPARγ phosphorylation, and increased PPARγ expression, inhibiting BACE1 expression and Aβ production. The PPARγ antagonist GW9662 blocked these pioglitazone effects (Quan et al., 2019b), affirming they were mediated by the PPARγ receptor.

PPARγ effects are dependent on the co-activator PGC-1α. Over-expression of PGC-1α in a cell line stably expressing APP inhibited Aβ production, concomitantly with decreasing BACE1 expression (Katsouri et al., 2011). Knocking out PPARγ expression abrogated the PGC-1α effect (Katsouri et al., 2011). The levels of both PPARγ and PGC-1α are reduced in brain extracts from Alzheimer’s cases compared with cognitively normal controls (Sastre et al., 2006; Qin et al., 2009; Katsouri et al., 2011). This is associated with reduced PPARγ binding to the BACE1 PPRE, and elevated Aβ production. By over-coming counter-regulatory effects of CDK5 and other signaling kinases, PPARγ agonist pioglitazone increases PPARγ expression, inhibiting BACE1 expression and blocking amyloid plaque formation.

We discuss NFTs here because of their ubiquitous association with amyloid plaques. Neurofibrillary tangles are correlated with neuronal dysfunction and brain atrophy more directly than are amyloid deposits (Brion, 1998; Jack et al., 2018). Pioglitazone inhibited tau phosphorylation (Cho et al., 2013; Hamano et al., 2016; Moosecker et al., 2019) and oligomerization (Hamano et al., 2016) in cell-based tauopathy models, and in pre-clinical mouse models (Escribano et al., 2010; Searcy et al., 2012). It also blocked misrouting of tau to dendritic spines *in vitro* (Moosecker et al., 2019). The PPARγ-specific antagonist GW9662 blocked these effects, confirming they were PPARγ receptor-dependent. Rosiglitazone was similarly effective in mice (Escribano et al., 2010). PPARγ preserves synapses, which may be due to the correction of tau’s mis-sorting (Moosecker et al., 2019). Pioglitazone also reduced tau phosphorylation in the 3xTg mouse AD model (Searcy et al., 2012). The effects on tau phosphorylation and aggregation may be a consequence of pioglitazone-mediated direct inhibition of CDK5 (Hanger et al., 1998). Additionally, PPARγ may regulate CDK5 indirectly through its effects on the inflammatory response. p35 is a regulatory protein that activates CDK5, and calpain-catalyzed cleavage of p35 in response to elevated cytosolic Ca2+ that occurs in neurons during the pathogenesis of AD cleaves p35 to form p25, which hyperactivates CDK5 and causes increased tau phosphorylation (Kimura et al., 2014; Seo et al., 2017). IL-6 enhances CDK5 activity (Quintanilla et al., 2004) by promoting the p35 – to – p25 conversion, and PPARγ suppresses IL-6 release (Jiang et al., 1998).

#### Late-Onset AD

Roughly 50% of the genes associated with late-onset Alzheimer’s encode proteins involved in the innate immune system, and many of the remaining genes encode proteins involved in lipid metabolism (Jones et al., 2010). Both Apolipoprotein E ε4 (*APOE* ε*4*), which is the most significant and highly replicated genetic risk factor for AD (Corder et al., 1993; Saunders et al., 1993; Lambert et al., 2013), and the *TREM2* R47H polymorphism, which has the second largest effect size (Guerreiro et al., 2013; Jonsson et al., 2013), affect innate immunity and lipid metabolism (Shi and Holtzman, 2018; Nugent et al., 2020). Metabolomics studies consistently point to pronounced alteration of lipid metabolism as an early marker of AD (Han, 2005, 2010).

*APOE* is one of a cluster of genes in the Chr 19q13.32 genomic region that affect AD risk, that also includes *PVRL2, TOMM40*, and *APOC1*. There are three common forms of *APOE*, distinguishable by the identity of amino acids at positions 112 and 158 that are determined by two closely linked SNPs in the *APOE* gene: rs429358 and rs7412, that result in the expression of three alternative protein isoforms: APOE ε2, which possesses cysteine residues at both positions, APOE ε3, which possess cysteine-112 and arginine-158, and APOE ε4, which possesses arginine residues at both positions. *APOE* ε*4* increases the risk for developing AD dose-dependently and also decreases the age of disease onset (Corder et al., 1993; Roses, 1994; Frisoni et al., 1995; Farrer et al., 1997). By contrast, *APOE* ε*2* is protective against AD, and *APOE* ε*3* has intermediate risk (Corder et al., 1994; Farrer et al., 1997). The brain produces all of its ApoE locally; the liver and macrophages produce peripheral ApoE. Glial cells account for most of the ApoE production in the brain. It mediates cholesterol and phospholipid transfer between astrocytes and microglia and neurons, on HDL-like lipoprotein particles. It is the main lipoprotein component of these particles, which are taken up by members of the low-density lipoprotein receptor family (Holtzman et al., 2012). Under conditions of stress, neurons also express *APOE* (Han et al., 1994a, b). Since the association between *APOE ε*4 and LOAD was first reported (Saunders et al., 1993), a variety of potential mechanisms underlying the contribution of *APOE* ε*4* to the pathogenesis of LOAD have been uncovered, from impaired neurite outgrowth (Holtzman et al., 1995), plasticity (Weeber et al., 2002) and repair (Ignatius et al., 1987), to defective Aβ clearance (Verghese et al., 2013; Kanekiyo et al., 2014; Mouchard et al., 2019), to mitochondrial dysfunction (Chen et al., 2011) and impaired endosome-lysosome trafficking (Nuriel et al., 2017; Zhao et al., 2017).

Although *APOE* is the most significant genetic risk factor for AD, it does not fully explain the risk attributable to the chr 19q13.32 genomic region. At least three additional genes in close proximity to *APOE*, *PVLR2, APOC1*, *TOMM40* (Takei et al., 2009; Roses, 2010; Zhou et al., 2019; Bussies et al., 2020; Fan K.H. et al., 2020; Squillario et al., 2020), make independent contributions.

*TOMM40* encodes the mitochondrial protein import channel, and is indispensable for maintaining mitochondrial homeostasis (Baker et al., 1990; Taylor et al., 2003) and for life (Zeh, 2013). Multiple SNPs in *TOMM40* are associated with AD risk independently of the *APOE* gene., including rs7259620 (Takei et al., 2009; Nazarian et al., 2019), rs760136 (Marioni et al., 2018), rs2075650 (He et al., 2016; Huang et al., 2016; Bussies et al., 2020; Soyal et al., 2020; Squillario et al., 2020), and rs10524523 (Roses, 2010; Li et al., 2013; Yu et al., 2017a, b). Both *APOC1* and *PVRL2* fit the pattern of being lipid- or immune-related. ApoC1 interferes with ApoE-mediated cholesterol and phospholipid uptake in the CNS by blocking the binding of ApoE-enriched lipoprotein particles to the low-density lipoprotein receptor (Kowal et al., 1990; Weisgraber et al., 1990; Sehayek and Eisenberg, 1991), and it blocks binding of APOE ε3- and APOE ε4-enriched particles equally well (Kowal et al., 1990). The *APOC1* risk haplotype was associated with plasma levels of Aβ40 (Zhou et al., 2019). *PVRL2* mediates the uptake of herpesvirus (Warner et al., 1998). There is enduring speculation that Herpes virus contributes to the etiology of AD (Itzhaki et al., 2016; Readhead et al., 2018), but this theory is controversial (Rizzo, 2020). The risk haplotype of *PCRL2* was associated with worsening cognitive performance, reduced total brain and hippocampal volume, and total serum Aβ42 (Zhou et al., 2019). Additionally, *PVRL2, APOE* and *APOC1* have regulatory roles on the expression of genes in this linkage disequilibrium region: *APOE* ε*4* suppresses the transcription of *TOMM40*, *APOE* and *APOC1* in the brain, while risk haplotypes of *PVRL2* and *APOC1* increase brain *APOE* expression, regardless of *APOE* genotype (Zhou et al., 2019). Methylation of the *TOMM40* promoter decreases expression of *TOMM40* and increases *APOE* expression (Shao et al., 2018). Together, these studies indicate that four genes in linkage disequilibrium on chromosome 19, *PVRL2, TOMM40, APOE* and *APOC1* independently affect brain structure, neuroenergetics and cognitive performance, and the risk for AD.

**FIGURE 3:**
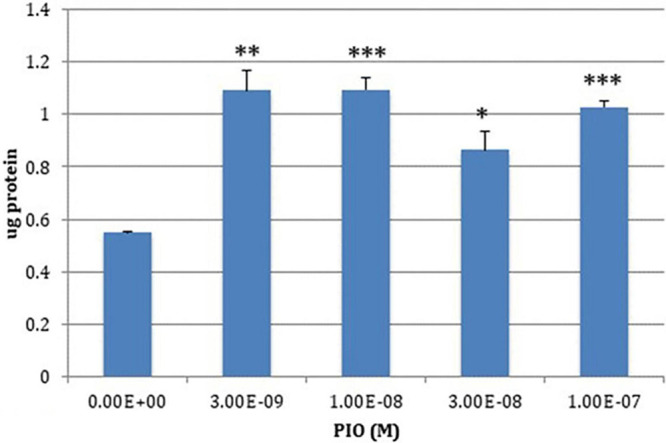
PIO-elicited TOM40 expression in SKNMC neuroblastoma cells. *P < 0.05; **P < 0.01; ***P < 0.001.

The chr 19q13.32 genomic region is enriched in PPARγ binding sites (Subramanian et al., 2017), which is not surprising since most endogenous PPAR ligands are lipids or lipid derivatives and the region is enriched in lipoproteins or proteins that interact with them (Zhou et al., 2019). PPARγ agonists affect the expression of three of the four genes in the region: *TOMM40*, *APOE* and *APOC1*; their effects on *PVRL2* expression have not been studied to date. Pioglitazone increases *APOE* expression in macrophages (reviewed in Ricote et al., 2004), and in the brain (Mandrekar-Colucci et al., 2012). By contrast, Subramanian et al. showed that reducing PPARγ expression in the human hepatoma line HepG2 paradoxically increased *TOMM40*, *APOE* and *APOC1* expression. Consistent with these results, low (nM) concentrations of pioglitazone suppressed expression of both *APOE* and *APOC1*, without detectable effects *TOMM40* expression. Other workers reported that high (μM)concentrations of the PPARγ agonists ciglitazone and 15d-PGJ2, elicited a robust increase in *APOE* expression and a modest suppression of *APOC1* expression (Dahabreh and Medh, 2012). These contrasting results may reflect the respective drug concentrations used, since bi-phasic PPARγ dose-effect curves have been reported (Wada et al., 2006; Miglio et al., 2009; Moon et al., 2012). Using the SKNMC cell line that is more pertinent to AD, we found that pioglitazone increased Tom40 protein expression (Charalambous et al., 2016; Figure 3).

### Lipid Metabolism

The pathology of AD is interwoven with extensive alterations in lipid metabolism (Foley, 2010), which are detectable in the CSF and plasma as well as the brain (Wood, 2012; Trushina et al., 2013; Varma et al., 2018). This topic has been reviewed recently (Penke et al., 2018; Kao et al., 2020), and we will limit our discussion to selected topics.

Ethanolamine plasmalogen (PIsEtn), comprises between 60 and 90 mol% of the total phospholipids of neuronal cell membrane fraction in human gray and white matter, respectively (Han, 2010), and over 60 mol% of all phosphatidylethanolamine in synaptic vesicles (Han, 2010). Plasmalogens are glycerophospholipids in which the substituent at the sn-1 position is a vinyl ether fatty alcohol (-O-CH = CH-R). They are protective against oxidative damage to polyunsaturated diacylphospholipids (Reiss et al., 1997), and they facilitate membrane fusion (Glaser and Gross, 1994). Plasmalogen deficiency is detectable at early stages of AD (Han et al., 2001). It is not detected in Huntington’s disease or Parkinson’s disease (Ginsberg et al., 1995; Farooqui et al., 1997). Although the deficiency is detected in both gray and white matter, it only worsens as AD pathology progresses in white matter (Han et al., 2001). Circulating levels of PIsEtn positively correlate with the extent of functional state deterioration (Wood et al., 2010). Very long chain fatty acids increase in late stages of AD, causing lipotoxicity (Schönfeld and Reiser, 2016) and exacerbating neuronal damage. Peroxisomes host both the synthesis of PIsEtn and the oxidation of very long-chain fatty acids, but they are deficient or dysfunctional in AD (Grimm et al., 2011; Kou et al., 2011). Pioglitazone corrects these defects, through several different mechanisms. As a partial PPARα agonist (Sakamoto et al., 2000; Orasanu et al., 2008), it promote peroxisome biosynthesis (Hoivik et al., 2004) and related lipid metabolism (Kersten, 2014). Pioglitazone also enhances PIsEtn synthesis by facilitating uptake of precursor fatty acids, including docosahexaenoic acid via fatty acid binding protein 5 (Pan et al., 2015). FABP5 knock-out mice exhibit impaired working memory and short-term memory (Pan et al., 2016), and pioglitazone increases FABP5 expression (Low et al., 2020). Pioglitazone also enhances PIsEtn synthesis through by inhibiting amyloidogenic processing of APP. The APP intracellular domain promotes expression of alkyl-dihydroxyacetone phosphate synthase (Grimm et al., 2011), the rate-limiting enzyme in PIsEtn synthesis. This fails when APP processing is diverted to the Aβ pathway, but pioglitazone blocks this and rescues PlsEtn synthesis.

Sphingolipids are major components of the myelin sheath, and MRI shows that demyelination occurs during the MCI phase (Bouhrara et al., 2018). Similarly, at early stages of pathogenesis, sulfatide sphingolipid levels are reduced by ∼ 92 mol% in gray matter, regardless of brain region, by 35 mol% in the cerebellum, and by 58 mol% in the temporal cortex. Rosiglitazone reversed myelin structural damage in a rodent model (Cowley et al., 2012).

Ceramide levels are ∼ 3-fold higher in white matter across all brain regions in MCI subjects vs. age-matched controls. The expression of an extensive gene network underlying ceramide synthesis also is increased at early stages of AD (Katsel et al., 2007). Increased availability of ceramides contributes to the pathogenesis of AD by causing mitochondrial damage and increasing apoptosis (Yu et al., 2000), and contributes to the depletion of PIsEtn via the stimulation of PIsEtn-PLA_2_ (Farooqui, 2010; Ong et al., 2010). Sphingosine-1-phosphates (S-1-P), metabolic products of ceramides, generally counteract ceramide effects (see Wang and Bieberich (2018) and Czubowicz et al. (2019) for excellent reviews). PPARγ (Parham et al., 2015) is one of several surface and intracellular receptors that mediate S-1-P effects and maintains the homeostatic phenotype in T-lymphocytes; this role has not been investigated in microglia.

## Innate Immunity

In significant measure, AD is a disease of the innate immune system (Zhang et al., 2013; Jones et al., 2015; Kan et al., 2015). Most (ca. 60%) of the AD GWAS at-risk genetic polymorphisms are in Sims et al. (2017) or near genes or their regulatory elements, that are enriched in microglia (Tansey et al., 2018; Jansen et al., 2019; Kunkle et al., 2019; Nott et al., 2019). *APOE* ε*4* expression and the expression of immune regulatory genes are positively correlated (Keren-Shaul et al., 2017; Mathys et al., 2019). Moreover, a haplotype associated with reduced expression of *PU.1*, a pivotal gene for microglial development (Turkistany and DeKoter, 2011), delays the age of onset of AD (Huang et al., 2017).

Microglia are the CNS’ resident innate immune system cells (Ginhoux et al., 2010; Schulz C. et al., 2012). Their primary function is to insure the health and connectivity of the neurons [Streit and Kincaid-Colton (1995) and Nayak et al. (2014) and references therein]. In their ‘quiescent’ state, microglia survey their local environments, including direct communication with neighboring neurons and astrocytes, through ramified extensions. Detection of specific signals generated by injury to the surrounding cells triggers activation of microglia, involving morphological transformations and triggering specific biochemical and genetic programs. To sustain the activated state, bioenergetic metabolism is switched from reliance on oxidative phosphorylation to glycolysis, which supplies not only ATP but also important metabolic intermediates including NADPH and other intermediates of the pentose phosphate shunt (Lauro and Limatola, 2020).

Microglia are exquisitely sensitive to deviations in their local environments and changes in microglial transcriptomics, morphology or behavior (phagocytosis) are often the first signs of pathology (Boza-Serrano et al., 2018). Their programmed transcriptional responses are bespoke for different stimuli, and support increased phagocytosis, the production of interferon and cytotoxic cytokines, chemokines, extracellular proteases and reactive oxygen species, as well as anti-inflammatory cytokines and factors that promote tissue repair and remodeling of the extracellular matrix (Porcheray et al., 2005; Stout et al., 2005). These inflammatory and immunosuppressive phenotypes represent the extremes of a spectrum of responses (Colton et al., 2006; Colton, 2009; Gray et al., 2020). Longitudinal gene transcription profiles of microglia isolated from mouse AD models reveal there are multiple discrete populations of activated microglia in the AD brain, reflecting interferon-related, proliferation-related and neurodegeneration-related phenotypes (Keren-Shaul et al., 2017; Friedman et al., 2018; Mathys et al., 2019).

Activated microglia play two distinct roles in amyloid metabolism. On the one hand, they promote the generation of Aβ peptides via interferon-mediated induction of IFITM3 (interferon-induced transmembrane protein 3). IFITM3, which previously had been recognized for its antiviral activity (Bailey et al., 2014), associates with the γ-secretase complex and promotes amyloidogenic APP processing (Hur et al., 2020). This role is consistent with the theory that Aβ peptides are part of the innate immune system’s anti-infection repertoire (Eimer et al., 2018). Activated microglia also participate in the clearance of amyloid deposits, forming clusters adjacent to – and sometimes surrounding – β-amyloid plaques (Condello et al., 2015). Some microglia associated with plaques become dysmorphic as the disease advances, through exhaustion or via collateral damage from unrestrained proinflammatory activity of adjacent microglia, and the plaques engulf some (Streit et al., 2009, 2018).

Increased expression of inflammation-associated genes, including *APOE*, and reduced expression of homeostatic genes characterize activated microglia. *APOE* may be required for the activation response (Ulrich et al., 2018). *APOE* expression is higher nearer the plaque (Krasemann et al., 2017), but the gradient signal is not known. ApoE suppresses expression of genes related to homeostatic microglia and reinforces proinflammatory gene expression (Krasemann et al., 2017) by activating NF-κB signaling (Ophir et al., 2005; Maezawa et al., 2006). NF-κB is a master regulator of the innate immune system and the inflammatory response (Liu T. et al., 2017). *APOE* ε*4* exacerbates these effects (Brown et al., 2002; Vitek et al., 2009; Zhu et al., 2012), perhaps by blocking differentiation to the immunosuppressive phase.

*TREM2* is part of the microglial surveillance system for monitoring changes in the environment, and regulating microglia responses to those changes, including proliferation, migration and activation. Variants of *TREM2* increase the genetic risk for late-onset AD 2 – 4X, which is second only to the effect size of *APOE* ε*4* (Jonsson et al., 2013; Guerreiro and Hardy, 2014). *TREM2* is a single-pass receptor that binds damage associated molecular signatures (DAMPS) (Daws et al., 2003), lipoproteins and lipoprotein particles, anionic lipids and sphingomyelins exposed by cellular damage, β-amyloid peptides (Wang et al., 2015; Yeh et al., 2016; Song W. et al., 2017). Ligand binding promotes association between the *TREM2* receptor with the adaptor protein *DAP12* (*TYROBP)*, which associate via electrostatic interactions, and activates an intracellular signaling cascade mediating the effects of ligand binding on survival and proliferation, phagocytosis and inflammation (Wang et al., 2015). In mouse AD models, *TREM2* mediated clustering of microglia around β-amyloid plaques and activation of phagocytosis and the ‘proinflammatory’ response (Jay et al., 2015; Wang et al., 2015; Ulrich et al., 2018; Zhao et al., 2018; Zhong et al., 2018), and was required for the full expression of the response to Aβ pathology across all microglial modules (Friedman et al., 2018). *APOE* and *TREM2* likely operate on the same molecular pathway because *APOE* is a ligand for *TREM2* (Atagi et al., 2015; Bailey et al., 2015; Jendresen et al., 2017), *TREM2* modulates the expression of almost all the genes in the core neurodegeneration bin except *APOE* (Friedman et al., 2018), and Trem2^–/–^ and Apoe^–/–^ mice are phenocopies (Ulland et al., 2017; Ulrich et al., 2018).

A R47H switch in the *TREM2* protein is the most common *TREM2* variant connected with AD. It is associated with increased total tau in the CSF, but does not affect the CSF Aβ peptide levels (Lill et al., 2015). *In vitro*, *APOE* bound to this *TREM2* variant with a lower affinity than to the wild-type receptor (without distinction between the *APOE* isoforms) (Atagi et al., 2015), and the R47H variant decreased the uptake of Aβ-lipoprotein complexes by monocyte-derived macrophages (Yeh et al., 2016).

Chemical dissection has shed additional light on the roles of microglia in AD and related tauopathies. TGF-1β and CSF-1 signaling sustain microglia, and *CSFR1* antagonists or cFMS inhibitors (Dagher et al., 2015; Spangenberg et al., 2016, 2019; Sosna et al., 2018), which deplete resident microglia from the brain, have been used as molecular scalpels. Contrary to expectations, eliminating microglia blocked the development of β-amyloid plaques and accumulation of intraneuronal amyloid, and it prevented the loss of neurons and synapses, and improved memory and learning. These salutary effects occurred whether the inhibitors were added early and maintained for long periods (Sosna et al., 2018; Spangenberg et al., 2019), or were added after plaque formation had reached advanced stages (Spangenberg et al., 2016). Blocking microglial proliferation with an inhibitor of the cFMS kinase, that autophosphorylates and activates the *CSFR*, similarly prevented the formation of amyloid plaques, improved memory and behavior and shifted the brain environment to an immunosuppressive phase (Olmos-Alonso et al., 2016). Hence, and contrary to expectations, microglia evidently are required for formation of amyloid plaques. Moreover, the absence of microglia or impaired microglia function is not detrimental for learning or memory. Shi et al. used the same strategy to learn the role of microglia in tau-mediated neurodegeneration. They demonstrated that microglial-mediated damage, and not tau-mediated toxicities, is responsible for neurodegeneration in a mouse tauopathy model (Shi et al., 2019). Therefore, it appears that microglial proliferation and/or activation is responsible for the neurodegeneration commonly associated with the two major pathologic hallmarks of Alzheimer’s disease, neurofibrillary tangles and β-amyloid plaques. It is possible that the microglial-mediated proinflammatory response, or the failure of the microglial immunosuppressive response, causes the damage leading to cognitive decline and dementia. Microglia are being targeted for neurodegenerative diseases (Dong et al., 2019).

### PPARγ and Innate Immunity

PPARγ agonists prime myeloid cells to respond to immunosuppressive stimuli and enhance the differentiation of myeloid cells into an immunosuppressive state (Bouhlel et al., 2007). PPARγ is widely distributed in mouse and human brain (Warden et al., 2016), including in microglia (Bernardo and Minghetti, 2006, 2008), and activation of myeloid cells with a proinflammatory stimulus increases *PPARγ* mRNA and protein expression (Fakhfouri et al., 2012; Song J. et al., 2017). PPARγ activators also increase *PPARγ* mRNA and protein expression. In myeloid cells, the PPARγ binding sites are adjacent to *PU.1* sites on macrophage/microglia-specific targets (Lefterova et al., 2010) and control the expression of *PU.1*-responsive genes (Lefterova et al., 2010). In addition to regulating the cytokines and cytokine receptors directly involved in the inflammation response, *PU.1* regulates expression of factors required for myeloid and lymphoid cell development (Turkistany and DeKoter, 2011), including *M-CSF* (Macrophage-specific CSF) (Zhang et al., 1994). PPARγ blocks *M-CSF* expression (Bonfield et al., 2008), and inhibits the transcription factors *AP-1*, *STAT3* and NF-kB (Ricote et al., 1998). Together with its effects on *PU.1*, the net result is suppression of pro-inflammatory activation and sensitization of microglia for differentiation into the immunosuppressive phenotype. Pioglitazone blocks the synthesis of pro-inflammatory molecules, including IL-1, TNFα, IL-6, iNOS, COX2, MMP9, and Caspase 3 (Kapadia et al., 2008) and promotes the synthesis of immunosuppression-related molecules, including Arg1, IL-4, IL-10, TGF*b*, catalase, SOD, and related genes (Bouhlel et al., 2007).

*In vitro* studies confirmed that PPARγ controls the cellular response to AD-related pathogenic triggers, including Aβ and LPS (Combs et al., 2000; Heneka et al., 2000; Hunter et al., 2008). These effects go beyond simply regulating expression of pro- and anti-inflammatory molecules. PPARγ overcomes pathogenesis-related developmental blocks that prevent transitioning of microglia to the immunosuppressive phenotype. iPSC-derived microglia that are heterozygous for the pathogenic *TREM2* R47H mutation have a shortfall in glycolytic capacity and cannot execute the metabolic switch that underpins differentiation of microglia to the immunosuppressive phenotype (Piers et al., 2020); consequently, they are deficient in phagocytosis. Pioglitazone corrected the glycolysis deficit, reversed blockade of the metabolic shift, and restored phagocytosis of Aβ42 (Piers et al., 2020). Pioglitazone achieved this by increasing phosphorylation and activation of p38-MAPK, which phosphorylated and activated MAPK2, which, in turn, phosphorylated and activated 6-phosphofructo-2-kinase/fructose-2,6-bisphosphatase 3 (PFKFB3), a key regulatory step in glycolysis.

Pioglitazone’s salutary effects on the innate immune system also correlated with a shift toward the immunosuppressive state in pre-clinical models of traumatic brain injury (Deng et al., 2020), depression (Zhao et al., 2016), axonal injury (Wen et al., 2018), neuroinflammation (Kielian and Drew, 2003) and stroke (Tureyen et al., 2007; Cai et al., 2018), and Parkinson’s disease (Swanson et al., 2011; Carta and Pisanu, 2013).

## Cerebral Glucose Homeostasis

The brain is dependent almost entirely on glucose for its energetics needs and consumes 25% of the body’s daily glucose load. In addition to energy production, glucose contributes to the synthesis of neurotransmitters, including acetylcholine, aspartate, glutamate, and GABA. In the fed state, neurons consume glucose directly, and glial cells, mostly astrocytes, store glucose as glycogen. Under oxidative stress, mitochondrial bioenergetics is compromised and neurons divert acetyl-CoA into fatty acids, which astrocytes take up and store as lipid droplets in an *APOE*-dependent process (Liu et al., 2015; Liu T. et al., 2017). These droplets are essential for neuronal health since defective transfer of lipids from neurons to astrocytes causes neurodegeneration (Liu L. et al., 2017). The droplets may represent essential energy reserves. During periods of normal fasting, astrocytes convert stored glycogen via glycogenolysis and glycolysis to lactate, which is consumed by neurons (Calì et al., 2019). Similarly, astrocytes may convert fatty acids stored in the lipid droplets to ketone bodies, for consumption by neurons. Under glucose insufficiency, neurons consume ketone bodies (Ding et al., 2013). Gene expression analysis of human AD subjects and mouse AD models reveal increasing reliance on lipid metabolism with disease progression as glucose consumption decreased (Yao et al., 2011; Demarest et al., 2020). Alternatively, the stored triglycerides may be used to synthesize membranes in support of phagocytosis, or in response to stress (Martínez et al., 2020). Finally, intracellular lipid droplets may be centers for coordinating glial-based responses to infectious agents, by attracting pathogenic microbes and acting as reservoirs for antimicrobial peptides and nucleation sites for other immune proteins, including RSAD2 (Bosch et al., 2020). Nor are these mutually exclusive options. [See Welte and Gould (2017) for a recent review of lipid droplets].

Glucose hypometabolism is a characteristic feature of Alzheimer’s disease. It is routinely measured by ^18^F-deoxyglucose-positron emission tomography (FDG-PET) (Minoshima et al., 1995, 1997; Herholz, 2010), or regional blood flow, measured by ^15^O-PET (Beason-Held et al., 2013), which are highly correlated. The sodium-insensitive GLUT1 and GLUT3 transporters account for most of the glucose extraction from the blood, and in persons with AD, the levels of these transporters in the brain begin to decline decades before the onset of AD symptoms (Simpson et al., 1994; Patching, 2017). GLUT1 is the predominant glucose transporter in the blood-brain barrier (BBB) and in astrocytes, and is responsible for the uptake from the systemic circulation of all of the glucose consumed by the brain. The high affinity, high capacity GLUT3 transporters are responsible for neuronal glucose uptake. The brain also expresses low levels of the insulin-sensitive GLUT4 transporter, in the cerebellum, cortex, hippocampus and hypothalamus, regions where the insulin receptor is also highly expressed (McEwen and Reagan, 2004; Alquier et al., 2006). Pioglitazone enhances nerve stimulation-coupled cerebral glucose uptake.

Reduced cerebral glucose utilization in AD is associated with reduced CSF levels of glycolytic intermediates (Bergau et al., 2019)reflecting impaired glycolysis and post-glycolytic pathways (An et al., 2018). It is independent of Aβ42 and Aβ40 levels (Venzi et al., 2017), or brain atrophy (Smith et al., 1992; Ibáñez et al., 1998) or other changes in brain structure (Small et al., 1995, 2000; Minoshima et al., 1997; Reiman et al., 2004; Samuraki et al., 2007; Beason-Held et al., 2013), and emerges decades before the appearance of clinical symptoms (Cutler, 1986; Kennedy et al., 1995; Small et al., 1995; Reiman et al., 1996, 2004; Beason-Held et al., 2013). It is associated with altered expression of energy metabolism genes in brain regions most vulnerable to AD pathology (Xu et al., 2006; Brooks et al., 2007; Wang et al., 2007, 2010; Liang et al., 2008a, b; Bossers et al., 2010), including the emergence of focal temporoparietal hypometabolism, which is distinct from normal aging (Kuhl et al., 1982; de Leon et al., 1983; Duara et al., 1984). Cerebral hypometabolism leads to increased tau phosphorylation (Planel et al., 2004) and amyloid accumulation (Gabuzda et al., 1994). Conversely, re-establishing homeostatic myeloid cell glucose metabolism by inhibition of the EP2 receptor reversed age-associated cognitive decline (Minhas et al., 2021). Thus, defective brain glucose metabolism is an early, consistent, and specific marker for neurodegeneration in AD that is consequential for and precedes AD pathology.

### Pioglitazone and Cerebral Glucose Metabolism

*In vivo*, pioglitazone improves cerebral blood flow and cerebral glucose uptake and disposal (Nicolakakis et al., 2008; Sato et al., 2011; Papadopoulos et al., 2013), in part via enhancing expression of the GLUT4 transporters (Sandouk et al., 1993; Olefsky and Saltiel, 2000), and in part by improving mitochondrial function and biogenesis. Pioglitazone also normalizes glucose metabolism by suppressing PGE2 synthesis and inhibiting PKA signaling that is triggered by EP2 (Subbaramaiah et al., 2012).

## Cerebral Insulin Resistance

Insulin resistance (Craft, 2005; Benedict et al., 2012; Willette et al., 2013, 2015a; Ferreira et al., 2018) and type 2 diabetes mellitus (DM2) (Chatterjee and Mudher, 2018; Barbiellini Amidei et al., 2021) are related but independent risk factors for AD. Both DM2 and cognitive impairment share gene expression networks that are enriched in genes involved in inflammation and PI3K-Akt signaling (Potashkin et al., 2019), and direct analysis of post-mortem brain samples revealed impaired insulin- and IGF1-triggered signaling in human and mouse AD brain samples (Bomfim et al., 2012; Talbot et al., 2012).

Insulin, insulin-like growth factor 1 and their respective mRNAs are found throughout the brain (Blázquez et al., 2014). The insulin receptor has been mapped to all cell types throughout the brain, with particularly high concentrations in the hippocampus and hypothalamus, and its roles in processes as diverse as systemic energy homeostasis (Chen et al., 2017), balance and movement (Zhao et al., 2004), and memory formation and consolidation (McNay et al., 2010; McNay and Recknagel, 2011; Kullmann et al., 2016) are well established.

The same gene encodes the brain and the peripheral insulin receptors (IR). However, the brain and peripheral receptors differ in three key ways. The brain receptor arises by alternate splicing of the IR gene and is smaller than the peripheral IR. Unlike the peripheral IR, insulin binding to the brain IR does not promote its internalization. Finally, the brain IR forms hybrid receptors with IGF1 receptors (IGF1R) more readily than the peripheral IR does. The IR and IGF1R receptors belong to the same receptor-tyrosine kinase family. Both are α2β2 heterotetramers, composed of two extracellular ligand-binding α-chains that are disulfide-linked to membrane-spanning β-subunits that possess tyrosine kinase activity. The α-chains are also disulfide-linked to each other. In general, the IR signal elicits metabolic responses, including the translocation of transporters from internal depots to the cell surface, and therefore stimulates glucose and amino acid uptake and metabolism, while the IGF1R predominantly affects protein synthesis and cellular growth. Activation of both receptors trigger changes in gene expression. Hybrid IR-IGF1R receptors are more abundant in the brain than in the periphery. As of this writing, the specific roles of these three receptor types in the CNS have not been resolved.

In the periphery, insulin primarily promotes glucose and lipid homeostasis. A key step is insulin-stimulated translocation of GLUT4 glucose transporters from intracellular pools to surface membranes in adipocyte and muscle, mediated by a cascade of signaling adaptor proteins and kinases that, via a chain of phosphorylations, connect successive kinases with target functional proteins that mediate vesicle translocation, protein synthesis, and activation of metabolic pathways. Ligand binding activates auto-phosphorylation of the receptor on tyrosine residues, creating binding sites for the adaptor proteins, IRS (IR, predominantly) or Shc (IGF1R, predominately), which, themselves, are tyrosine phosphorylated. These form the hubs for signaling cascades. Two cascades stem from the IRS hub: the PI3K-Akt pathway promotes the translocation of transporters from intracellular depots to the cell surface and is responsible for the metabolic effects of insulin. The MAPK pathway, which both IRS and Shc control, mediates insulin’s (and IGF1’s) effects on gene expression. Both pathways cooperate in regulating cellular growth, differentiation and repair (Boucher et al., 2014).

In the brain, the insulin transduction pathway not only promotes trafficking of GLUT4, but also of the high affinity choline transporter and the AMPA, NMDA and GABA receptors (Zhao et al., 2004; Fishwick and Rylett, 2015; Spinelli et al., 2019). In addition to these post-synaptic effects, insulin promotes dendritic spine and synapse formation (Lee et al., 2011). Insulin does not regulate most of the brain’s glucose consumption, because GLUT1, the main glucose transporter of the BBB, and GLUT3, the main glucose transporter within the brain, are not insulin responsive (Simpson et al., 2008). However, insulin does stimulate glucose utilization in the hippocampus, one of the few brain regions that express GLUT4 transporters. To meet the high energy demands associated with memory formation and retrieval, translocation of the GLUT4 transporters is also mediated by AMPK under the control of the membrane potential (Ashrafi et al., 2017). Both the IR and GLUT4 are concentrated in the synapses, and insulin-stimulated, GLUT4-mediated glucose uptake supports sustained synaptic vesicle recycling (Ashrafi et al., 2017), and is essential for memory formation (Pearson-Leary and McNay, 2016; Pearson-Leary et al., 2018). Post-translational modification of mitochondria by the glucose sensor N-acetylglucosamine O-transferase localize the mitochondria within the same cellular regions as the IR and GLUT4 (Pekkurnaz et al., 2014). O-GlcNAcylation is also required for full activity of the mitochondrial ATP synthase (Cha et al., 2015). The co-localization of insulin receptors with GLUT4 transporters and mitochondria underscore the importance of insulin regulated glucose uptake and metabolism for supporting the energetic requirements of synaptic vesicle trafficking and the action potential. Tau is also O-GlcNAcylated under homeostatic conditions, but insulin resistance perturbs O-GlcNAc cycling and contributes to tau hyperphosphorylation (Liu et al., 2009, 2011; Bourré et al., 2018).

It is clear that, from facilitating the synthesis of acetylcholine at two levels (acetyl-CoA generation via the sequential action of glycolysis and pyruvate dehydrogenase, and choline uptake), to manipulating neurotransmitter release and uptake, to supporting neuritogenesis and repair, and regulating tau phosphorylation, insulin has profound effects on the processes that support cognition.

Given the important role insulin plays in brain physiology, it is not surprising that cerebral hypoinsulinemia, caused by peripheral insulin resistance, or cerebral insulin resistance *per se* are an important risk factors for neurodegenerative diseases, including Alzheimer’s disease (Baura et al., 1996; Matsuzaki et al., 2010; Willette et al., 2015b, c; Ekblad et al., 2017; Kong et al., 2018). Insulin resistance in mid-life predicts dementia in late life (Ekblad et al., 2017, 2018; Lutski et al., 2017; Tortelli et al., 2017; Kong et al., 2018). Increasing metabolic control with (Ryan et al., 2006) or without pharmacological intervention (Naor et al., 1997) improves working memory. DM2, which reflects systemic insulin resistance coupled with pancreatic insufficiency, is a risk factor for AD (Ott et al., 1999; Schrijvers et al., 2010; Livingston et al., 2020). The Metabolic Syndrome, reflecting systemic insulin resistance in conjunction with lipid and cardiac co-morbidities, also is associated with AD, independently of the *APOE* genotype (Kuusisto et al., 1997). While these relationships reflect systemic insulin resistance, it is critical to note that brain tissue itself is insulin resistant in AD in subjects who, at the time of death, were without other co-morbidities that feature insulin resistance, such as DM2, obesity or the metabolic syndrome. AD-associated brain insulin resistance is detectable early in life, in high-risk individuals who were not cognitively impaired at death. Reductions in insulin and IGF-1 signaling are detectable by inhibitory phosphorylation of insulin signaling-related proteins *in situ*, by the reduced activities of kinases in the insulin-signaling cascade, by impaired activation of the insulin-signaling cascade *ex vivo* (Steen et al., 2005; Bomfim et al., 2012; Talbot et al., 2012), and by dysregulated expression of genes encoding members of the insulin/IGF1 signaling pathways (Katsel et al., 2018). Deficiencies in insulin signaling are additive and were greater in individuals who suffered from both AD and DM2 (Liu et al., 2011). Mouse EOAD models also exhibit defective insulin signaling (Takeda et al., 2010; Bomfim et al., 2012). For excellent recent reviews of the association of brain insulin resistance with Alzheimer’s disease, see Ferreira et al. (2018), de la Monte (2019).

Many of the determinants contributing to AD trigger and sustain brain insulin resistance. Proinflammatory cytokines, including IL-1β and TNFα (Geng et al., 1996; Morrison et al., 2010; Bomfim et al., 2012; Kitanaka et al., 2019) activate ‘counter regulatory’ kinases such as ERK2, JNK and PKCζ/λ(20), which disrupt insulin/IGF1 – PI3K – Akt signaling. They phosphorylate proteins in the IR/IGFR signaling cascade at sites that interfere with normal docking or kinase activity. β-amyloid fibrils and oxidative stress consequent to mitochondrial dysfunction also activate these kinases (Okazawa and Estus, 2002; Persiyantseva et al., 2013). Inflammation and oxidative stress similarly account for systemic insulin resistance (Czech, 2017).

### Pioglitazone Overcomes Cerebral Insulin Resistance

One-significant way pioglitazone reduces the risk for AD is by overcoming cerebral insulin resistance and enhancing blood flow and glucose uptake/utilization. It overcomes each of the drivers behind brain insulin resistance: It restores normal expression of genes of the insulin signaling pathway (Katsel et al., 2018) and promotes glucose uptake in vulnerable neurons, it reduces inflammation and promotes immunosuppression (Zhang et al., 2008; Haraguchi et al., 2008; Swanson et al., 2011; Kaplan et al., 2014), it ameliorates oxidative stress (Gumieniczek, 2003; Wang et al., 2014; Paciello et al., 2018), and blocks the synthesis of Aβ peptides (Liu et al., 2013; Quan et al., 2019b) and promotes their rapid clearance from the brain (Mandrekar-Colucci et al., 2012). By overcoming systemic insulin resistance, including in subjects who are not diabetic, pioglitazone also relieves cerebral hypoinsulinemia (Baura et al., 1996; Miyazaki et al., 2002; Kernan et al., 2003).

## Mitochondrial Dysfunction

### Bioenergetics

Mitochondrial dysfunction is a major contributing factor to defective cerebral energy metabolism in AD. Oxidative damage and mitochondrial stress rank among the earliest detectable events in human AD (Hirai et al., 2001; Nunomura et al., 2001; Sultana and Butterfield, 2009), and mouse models (Yao et al., 2009). Altered mitochondrial morphology is evident in dendritic profiles, spines and synaptic terminals, and in astrocytes throughout the brain (Baloyannis, 2011), and belies bioenergetic defects (Parker et al., 1994a; Valla et al., 2001, 2010; Yao et al., 2009), due in part to reduced expression of nuclear-encoded mitochondrial genes and faulty repair of mtDNA defects (Lovell et al., 2000; Weissman et al., 2007; Sykora et al., 2015), and impaired dynamics (Manczak et al., 2011), and proteostasis (Alikhani et al., 2011; Westerlund et al., 2011). Inhibition of mitochondrial energy production elicits amyloidogenic processing of APP (Gabuzda et al., 1994) that, in turn, worsens mitochondrial function (Manczak et al., 2006; Cenini et al., 2016). Swerdlow (2018); Wang et al. (2020) and others (Lin and Beal, 2006; Reddy and Beal, 2008; Gibson et al., 2010; Moreira et al., 2010; Swerdlow et al., 2010, 2014) have thoroughly reviewed the contribution of mitochondrial damage to the pathogenesis of Alzheimer’s, and we note the highlights here.

Although mitochondria possess their own genomes, they encode only 13 of the ∼1500 mitochondrial proteins. The remainder are encoded by the nuclear genome, and the expression of many of these nuclear-encoded mitochondrial genes is dysregulated in early AD. The pattern of disruption roughly parallels the gradient of brain regions that exhibit hypometabolism (Liang et al., 2008b), from the posterior cingulate cortex (PCC), which is severely affected, to the middle temporal gyrus, the hippocampus, the entorhinal cortex, the visual cortex, and the superior frontal gyrus which is relatively spared from metabolic abnormalities (Minoshima et al., 1997; Mosconi et al., 2008b; Herholz, 2010). In addition to mitochondrial genes, glycolytic and TCA pathway genes also are down-regulated in AD (Brooks et al., 2007).

TOMM and TIMM encode components of the outer and inner mitochondrial membrane complexes, respectively, that catalyze import of nuclear-encoded mitochondrial proteins (Wiedemann et al., 2004). In addition to their importance for mitochondrial health (Zeh, 2013), they are critical for controlling cytosolic proteostasis (Liu W. et al., 2018) because blocked importation leads to excessive cytosolic accumulation of misfolded proteins. Mitochondrial protein import is dysregulated in AD (Anandatheerthavarada et al., 2003; Devi and Anandatheerthavarada, 2010; Devi and Ohno, 2012; Cenini et al., 2016; Sorrentino et al., 2017), and this dysregulation follows the same regional pattern as for OXPHOS genes. In AD, 50% of TOM complex proteins and 27% of the TIM proteins were under-expressed in the posterior cingulate cortex, but their expression was reduced by only 17% and 0%, respectively, in the visual cortex (Liang et al., 2008b).

The expression of subunits of each of the five complexes comprising the OXPHOS system is also inhibited in AD, and the extent of under expression in each brain region was consistent with the expression patterns of the respective mRNAs. These studies compliment earlier reports of reduced activity in AD brain of COX (Parker et al., 1994a; Gonzalez-Lima et al., 1997; Bosetti et al., 2002), α-ketoglutaric acid dehydrogenase (Gibson et al., 1988; Bubber et al., 2005) and pyruvate dehydrogenase (Sorbi et al., 1983; Rex Sheu et al., 1985). COX activity also is reduced in blood platelets in AD (Parker et al., 1994b; Bosetti et al., 2002; Valla et al., 2006), suggesting that mitochondrial-related AD pathophysiological determinants are not restricted to the brain.

While these data provide a biochemical rationale for AD-related cerebral hypometabolism, it is possible the observed mitochondrial deficits resulted from AD-related damage. Valla et al. tested this hypothesis in expired young adult *APOE* ε*4* carriers, who were at risk for developing AD, and in age-matched controls lacking *APOE* ε*4* (Valla et al., 2010). There were no histologic β-amyloid deposits, neurofibrillary tangles, or soluble Aβ42 in either group, and they were matched for insoluble Aβ42 and soluble Aβ40. None-the-less, the activity and protein levels of COX were lower in the at-risk population, confirming mitochondrial damage occurs prior to detectable pathological signs of AD.

The data summarizing mitochondrial dysfunction in AD and the conceptual picture it has engendered are based on autopsy specimens because it has not been possible to probe mitochondria in living subjects. Rather, mitochondrial function in living subjects has been inferred from FDG-PET analysis, which measures glycolysis directly. Tsukada’s group have now introduced a PET ligand that binds to the rotenone-inhibitable site on the mitochondrial OXPHOS complex I, and provides a direct measure of complex I availability in the living brain (Harada et al., 2013; Terada et al., 2020). They discovered the loss of complex I precedes FDG-PET-detectable hypometabolism in the parahippocampus in AD (Terada et al., 2020), confirming that mitochondrial dysfunction is an early event in AD pathogenesis. Mitochondria-related markers in the CSF offer additional tools for directly probing brain mitochondrial health. Podlesniy et al. showed that mtDNA levels in CSF are lower in asymptomatic subjects who are at risk for developing AD and in AD patients relative to cognitively normal, age-matched controls (Podlesniy et al., 2013). This defect was not observed in subjects with frontotemporal lobar degeneration (FTLD), which is nosogenically related to Alzheimer’s (Podlesniy et al., 2013). Previously, reductions in mtDNA in AD brain were detected by immunohistochemistry of whole brain (Hirai et al., 2001), and qPCR in single cells isolated by laser capture microdissection (Rice et al., 2014). These *post mortem* data support the use of CSF mtDNA quantification as an *in vivo* measure of mitochondrial health.

### Mitochondrial Dynamics

Synaptic loss is evident early in AD pathogenesis and is highly correlated with the severity of AD-related cognitive defects (DeKosky and Scheff, 1990; Terry et al., 1991). Mitochondria are highly dynamic organelles, and are continually redistributing within cells to meet specific regional needs, and undergoing shape changes and continuous and simultaneous rounds of fission and fusion necessary for maintaining functional mitochondria. In healthy neurons, mitochondria are uniformly distributed throughout the neuron, in both the cell body and in synapses, and support synaptogenesis and synapse function via their roles in signal transduction (Werner and Werb, 2002; Yang et al., 2009), ATP production (Rangaraju et al., 2014; Sobieski et al., 2017) and Ca^2+^ -buffering (Contreras et al., 2010; Tarasov et al., 2012). By contrast, in neurons from AD subjects, mitochondria are largely restricted to the cell bodies (Baloyannis et al., 2004; Wang et al., 2009; Pickett et al., 2018). Both APP and tau contribute to disrupted mitochondrial distribution. Axonal trafficking of mitochondria was retarded in *APP/PS1* neurons, and neurons from *PS1* and Tg2576 *APP* mice (Calkins et al., 2011; Trushina et al., 2012). Exposing neurons isolated from *APP* over-expressing mice to Aβ42 in culture inhibited mitochondrial trafficking and reduced axonal mitochondrial density (Du et al., 2010). Exposing hippocampal neurons from control C57Bl/6 mice to the Aβ25-32 peptide produced similar results (Calkins and Reddy, 2011). Disease-associated tau mutations, including hyperphosphorylated tau and the P301L mutation, disrupt interactions between microtubules and cargo, including mitochondria, and impede normal trafficking (Kopeikina et al., 2011; Schulz K.L. et al., 2012; Shahpasand et al., 2012; Rodríguez-Martín et al., 2016). Depletion of tau protects against Aβ-elicited mitochondrial trafficking deficits (Vossel et al., 2010). This may contribute to the mitigation of neuronal dysfunction observed in tau knock-down APP mice (Roberson Scearce-Levie et al., 2007; Ittner et al., 2010). Insulin resistance and inhibition of O-GlcNAc synthesis, which is necessary for the synaptic tethering of mitochondria (Pekkurnaz et al., 2014), may also disrupt the distribution of mitochondria in neurons.

Impaired mitochondrial dynamics also contributes to mitochondrial functional defects. It is associated with defective expression of the mitochondrial fission genes *DRP1* and *FIS1*, and the mitochondrial fusion genes *MFN1*, *MFN2* and *OPA1* (Wang et al., 2009; Manczak et al., 2011). *FIS1* expression is enhanced, and *MFN1*, M*FN2* and *OPA1* expression is suppressed in brain samples from human AD subjects compared with cognitively normal, age-matched controls. However, while Manczak et al. (2011) reported elevated *DRP1* expression in AD samples, Wang et al. reported decreased Drpt protein expression in AD without a change in its mRNA expression (Wang et al., 2009). These differences could reflect differences in the AD samples (including Braak stage and *APOE* status) used by these investigative teams. The distribution of the fission and fusion proteins matched that of mitochondria in brains from healthy vs AD cases, respectively (Wang et al., 2009). The GTPase activity of Drp1 is enhanced by phosphorylation on S616 (Taguchi et al., 2007). Western blot analysis revealed greater Drp1 S616 phosphorylation in both the mitochondrial and cytosolic fractions from AD subjects than from age-matched, cognitively normal controls (Wang et al., 2009); thus, even though Wang et al. observed lower levels of total Drp1 protein in AD, they detected higher levels of phosphorylated, and presumably activated, Drp1. Amyloid peptides or oligomers may activate Drp1. By co-immunoprecipitation and IHC, Manczak et al. showed monomeric and oligomeric Aβ physically associated with Drp1, which increased with increasing severity of disease. Over-expression of the APPswe mutation in neuroblastoma cells or in primary cultured neurons increased mitochondrial fragmentation and a perinuclear distribution of mitochondria, which was reversed by a BACE inhibitor reversed (Wang et al., 2008). Wang et al. subsequently showed that exposure of cultured neuroblastoma cells to oligomeric Aβ increased Drp1 phosphorylation and accumulation in the mitochondrial fraction, and mitochondrial fragmentation (Wang et al., 2009). Tau also interacts physically with Drp1, in a way that may increase Drp1 GTPase activity. This association has been detected in human AD frontal cortex, but not in controls, and was confirmed in cortical samples from *APP*, *APP/PS1* and 3 x Tg mice, but not in age-matched littermate controls (Manczak and Reddy, 2012).

These results suggest Drp1 activity, or its association with amyloid and/or tau, might be attractive targets for delaying or treating AD. Kuruva et al. used molecular docking simulations to design DDQ, that blocks binding of Drp1 with amyloid (Kuruva et al., 2017). In cultured neuroblastoma cells, DDQ blocked association of Aβ with Drp1, prevented mitochondrial fragmentation and oxidative stress, and enhanced mitochondrial biogenesis and synaptogenesis (Kuruva et al., 2017). We propose that pioglitazone also checks Aβ- and tau-mediated Drp1 activation, by virtue of its effects on Aβ production and tau phosphorylation.

Cells use a series of integrated pathways involving crosstalk among all the major organelles to control the synthesis, folding and trafficking of proteins. Dysfunction of any branch in any compartment has system-wide repercussions. Many cytosolic proteins that are prone to aggregation are imported into mitochondria and degraded (Ruan et al., 2017). Aβ peptides can be imported into mitochondria via the TOM complex (Hansson Petersen et al., 2008), using TOMM20 as the importation ‘receptor’(Hu et al., 2018). Overwhelming the mitochondrial proteostasis system with Aβ peptides leads to a number of adverse consequences, including: increased oxidative stress via the generation of ROS that is produced following inhibition of a fatty acid short chain dehydrogenase/reductase (Lustbader et al., 2004) and of OXPHOS complex I (Bobba et al., 2013), inhibition of mitochondrial trafficking and reduced axonal mitochondrial density (Du et al., 2010; Calkins and Reddy, 2011), and increased mitochondrial fragmentation (Baloyannis, 2006). If intramitochondrial degradation of Aβ is impaired (Lautenschläger et al., 2020), or if importation into mitochondria is blocked (Liu Y. et al., 2018), cytosolic proteostasis becomes blocked, leading to protein accumulation and aggregation in the cytosol (reviewed in Lautenschäger and Schierle, 2019). The fact that two proteases involved in mitochondrial protein homeostasis, PreP (Alikhani et al., 2011) and Htr2 (Westerlund et al., 2011), are reduced in AD, and the suggestive evidence that polymorphisms in Htr2 are associated with AD (Westerlund et al., 2011), are consistent with this model. However, the relationship between mitochondrial Aβ accumulation and extramitochondrial protein aggregation is non-linear because Aβ-mediated inhibition of bioenergetics and the processing of APP are related by a feedback cycle, wherein Aβ-mediated inhibition of bioenergetics stalls APP processing and the generation of additional β-amyloid (Wilkins and Swerdlow, 2017). Nonetheless, the intracellular accumulation and mitochondrial localization of both β-amyloid and phospho-tau, contribute to mitochondrial dysfunction and aberrant trafficking and dynamics that characterizes Alzheimer’s disease. By inhibiting the generation of Aβ peptides and the phosphorylation of tau, pioglitazone blocks these effects.

### Mitochondrial and Cellular Calcium Dysregulation

Calcium homeostasis is perturbed in AD (Khachaturian, 1994), which may contribute to both early and late (Bezprozvanny and Mattson, 2008; Calvo-Rodriguez et al., 2020) phases of the disease. Calcium is essential for multiple neuronal activities in addition to mitochondrial function, including neuritogenesis and synapse formation, synaptic transmission and synaptic plasticity. Altered calcium homeostasis is a cardinal feature of Alzheimer’s and other neurodegenerative diseases (Mattson, 2007; Tong et al., 2018), due, in part, to extrinsic factors, such as β-amyloid accumulation (Bezprozvanny and Mattson, 2008), but also to intrinsic factors. Increased influx through the voltage gated calcium channel and exaggerated calcium release from the ER, coupled with blunted reuptake (Popugaeva and Bezprozvanny, 2013), elevates cytosolic calcium (Thibault et al., 2007). These contribute to mitochondrial calcium overload (Calvo-Rodriguez et al., 2020), contributing to excess ROS production and impaired mitochondrial energy production and apoptosis (Cenini and Voos, 2019). Elevated cytosolic Ca2^+^ also activates CDK5 via calpain, leading to generation of the hyperactive p25 regulatory subunit (Kimura et al., 2014; Seo et al., 2017). The breakdown of neuronal calcium homeostasis extends to expression of genes important in calcium regulation (Emilsson et al., 2006). Oxidative stress and lipid peroxidation (Mattson, 1998), perturbations in the mitochondrial-ER membrane (MAM) (Hedskog et al., 2013; Area-Gomez et al., 2018) and the accumulation of β-amyloid peptides (Mattson et al., 1992) contribute to altered calcium homeostasis in AD.

Taken together, morphological analysis and gene expression, proteomic and functional data all support the conclusion that mitochondrial dysfunction is present early, before the detectible stages of AD pathology, including the accumulation of Aβ plaques or tau tangles, and contributes to AD pathogenesis.

### Pioglitazone and Mitochondrial Dysfunction

PPARγ agonists ameliorate AD-related mitochondrial dysfunction by inhibiting Aβ peptide production, discussed above, by eliciting mitobiogenesis (Strum et al., 2007; Miglio et al., 2009), and improving mitochondrial membrane potential (Wang et al., 2002; Pipatpiboon et al., 2012). They also limit oxidative stress damage, by inhibiting ROS generation by complex I (Brunmair et al., 2004; Ghosh et al., 2007) and by increasing expression of glutathione and the antioxidants SOD and catalase (Collino et al., 2006; Aleshin and Reiser, 2013). PPARγ agonists exert additional positive effects on neuronal energy balance by stimulating GLUT3 expression (Garcia-Bueno et al., 2006; Wang et al., 2012), which is decreased in AD (Simpson et al., 1994), by stimulating GLUT4-mediated glucose uptake and by promoting neuronal lactate oxidation (Izawa et al., 2009) and pyruvate flux (Rossi et al., 2020) via enhancing insulin-stimulated Akt activation (Karwi et al., 2020) and inhibiting PDH kinase activity (Way et al., 2001).

## Preclinical Efficacy of Pioglitazone and Rosiglitazone

Table 1 summarizes representative pre-clinical studies involving the PPARg agonists rosiglitazone or pioglitazone. This is not meant to be an exhaustive list, but the studies were selected to illustrate several points. First, both drugs exhibit *in vivo* efficacy on at least some AD-related phenotypes, but not all study results could be replicated. Generally, PPARγ agonists protected against oxidative damage, promoted synapse recovery and improved learning and memory, enhanced cerebral blood flow and glucose uptake, reduced corticosterone levels and amyloid deposits, Aβ peptide levels and reactive astrocytes and microglia, and promoted microglial phagocytosis. Two important generalizations can be drawn from these studies, that are important for considering the appropriate design of human trials.

**TABLE 1:** Summary of PPARγ agonist effects on pre-clinical models of Alzheimer’s disease.

**Model/references**	**Dosing**	**Human Equivalent Dose^a^**	**Comments**	**Results**
***Treatment paradigm***				
Tg2576 (Pedersen et al., 2006)	Rosiglitazone, 4 mg/kg, po (chow), vs vehicle control, 4 months.	19.51 mg/d	Male, 9 months old at initiation of treatment; amyloid deposits, hippocampal dendrite spine loss, and defective spatial learning evident when treatment initiated.	Rosiglitazone was statistically associated with improved memory, reduced learning deficits (radial arm maze); reduced insoluble Aβ42 levels; reduced corticosterone levels.
Tg2576, Nenov 2014, 2015 (Nenov et al., 2014, 2015)	Rosiglitazone, 30 mg/kg, po (chow), vs vehicle control, for 30 days.	146.35 mg/d	Equal numbers of males and females, 8 months old at initiation of treatment.	Learning, memory improvements correlated with improved spontaneous synaptic activity and short-term plasticity; engagement of the ERK pathway and expression of synaptic proteins, restoration of mature: immature DG granule cell ratio, normalized Na_*v*_-mediated currents.
*APP (V717I)*, (Heneka et al., 2005)	Pioglitazone, 40 mg/kg/d; po (chow), vs vehicle control, 7 days	195.12 mg/d	Equal numbers of males and females; 10 months old at initiation of treatment; amyloid pathology present.	Decreased BACE1, amyloid plaque deposits, soluble Aβ42 levels, reactive microglia.
*APP (Swe/PS1)Δ9*, (Mandrekar-Colucci et al., 2012; Skerrett et al., 2015)	Pioglitazone, 80 mg/kd/d, po (gavage) vs vehicle control, 9 days	390.24 mg/d	Equal numbers of males and females; similar results for mice that were 6 or 12 months-old at initiation of treatment and that exhibited differing pathological loads.	Improved memory retention. Decreased amyloid plaque deposits, reduced soluble and insoluble Aβ_40_ and Aβ_42_ in 6-month-old mice; reduced insoluble Aβ42 and Aβ40 and soluble Aβ40 in 12-month-old mice; reduced reactive microglia and astrocytes and enhanced microglial phagocytosis; increased IL-1β, TNFα, Tm1, Fizz1, Arg1 expression;
*APP/PSI* (Chen et al., 2015)	Pioglitazone, 10 mg/kg/d, ip, vs. vehicle control, 7 and 10 days.	48.78 mg/d	Equal numbers of males and females; 12 months old at initiation of treatment; pathology present.	Improved LTP after 7 days treatment & water maze performance after 10 days. Reduced CDK5 expression and activity as tau phosphorylation surrogate.
APP (Swe/PS1)Δ9 (Toba et al., 2016)	Pioglitazone, 80 mg/kg/d, po (chow), vs vehicle control, 9 days.	390.25 mg/d	Equal numbers of male and female mice, 5 – 6 months old at initiation of treatment, emergent stages of pathology.	Increased motor coordination, LTP; decreased CDK5 regulatory protein (p25 & p35) expression.
J20 (V717F under PDGF promoter) (Escribano et al., 2010)	Rosiglitazone, 5 mg/kg/d, po (gavage) vs vehicle control, treated for 1 month and 4 months.	24.39 mg/d	Equal numbers of males and females; 10 months old at initiation of treatment.	Improved object recognition after one month and progressively improved spatial memory (Morris Water Maze); reduced amyloid plaque and insoluble Aβ42 and Aβ40 levels, and phosphorylated tau, and promoted anti-inflammatory, pro-phagocytic microglial phenotype.
J20 (Nicolakakis et al., 2008)	Pioglitazone, 20 mg/kg/d, po (chow), vs vehicle control, for 1.5 – 2 months.	97.56 mg/d	Equal numbers of males and females; 14 months old at initiation of treatment; amyloidosis, neuronal loss well established.	No effect on water maze performance, amyloid deposits or levels of soluble or insoluble Aβ42 or Aβ40. Improved cerebral blood flow and glucose uptake; restored cerebrovascular function; trend toward improved cortical cholinergic stimulation; reduced astrogliosis; reversed cerebral oxidative stress.
3xTg, Search (Searcy et al., 2012)	Pioglitazone, 18 mg/kg, po (chow), vs vehicle control, for 3.5 months	87.81 mg/d	Female mice, 11 – 12 months of age when treatment initiated; amyloid deposits well-established, tau aggregates present.	Improved learning on the active avoidance task; enhanced LTP; reduced amyloid deposits and hyperphosphorylated tau in CA1.
***Prevention paradigm***				
J20 (Escribano et al., 2009)	Rosiglitazone, 5 mg/kg/d, po (gavage) vs vehicle control.	24.39 mg/d	Prevention vs. rescue study. Equal numbers of 1.5-month-old males and females were treated for 2.5 months (prevention), and equal numbers of 9-month-old mice were treated for 1 month (rescue).	Improved object recognition in both cohorts. In older mice, reduced corticosterone levels and blocked glucocorticoid receptor down-regulation.
J20 (Badhwar et al., 2013)	Pioglitazone, 20 mg/kg/day, po (chow) vs. control	97.56 mg/d	Treatment initiated with 3-month-old mice for 14 weeks; small cohort for 3 days.	14-week treatment that was initiated in young mice was statistically associated with improved spatial learning, with trend toward improved memory. Three-day treatment rescued cerebral blood flow; effect persisted in the longer-term treated mice.
Tg2576 (Rodriguez-Rivera et al., 2011)	Rosiglitazone, 30 mg/kg, po(chow), vs vehicle control, for 4, 8 or 12 months	146.16 mg/d	Equal numbers of males and females, 1 month old at initiation of treatment.	Reversed associative learning and memory deficits in 9-month old animals (fed for 8 months), but not 5 (fed for 4 months) or 13 (fed for 12 months) month-old mice.
SCAMP8 (Seok et al., 2019) SCAMP8 is a spontaneous ‘Alzheimer’s-like’ mouse model that exhibits amyloid and tau pathology, neuron and dendrite spine loss, and CNS oxidative stress (Armbrecht et al., 2014; Cheng et al., 2014)	Pioglitazone, 2 or 5 mg/kg/d, po (gavage) versus vehicle control, for 7 weeks.	9.76 or 24.39 mg/d	Equal numbers of male and female mice, 9 months old at initiation of treatment.	Improved water maze performance, reduced amyloid deposits and soluble Aβ40; increased LRP1 expression. All responses were attenuated at 5 mg/kg/d vs 2 mg/kg/d.
***Cerebrovascular model***				
J20/TGFβ1 (Papadopoulos et al., 2013)	Pioglitazone, 20 mg/kg/d, po (chow), vs vehicle control, 6 months	97.56 mg/d	Also a ‘treatment vs prevention study.’ Equal numbers of males and females. Two cohorts, 6 and 12 months of age at beginning of treatment. Adult mice treated for 6 months; aged mice treated for 3 months.	No effect on spatial learning or memory; improved reversal learning in adult but not aged mice. In both adult and aged cohorts, improved cerebral blood flow, cerebral glucose uptake; suppressed astrogliosis in cortex but not in hippocampus; suppressed microglial activation in hippocampus. No effect in either cohort on amyloid pathology, or on cerebrovascular reactivity.
TGFβ1 (Lacombe et al., 2004)	Pioglitazone, 18 mg/kg/d, po (chow), vs vehicle control, 2 months.	87.81 mg/d	Equal numbers of male and female mice, 2 months old at initiation of treatment.	Decreased Aβ42 levels and glia activation, and increased hydrocephalus.
TGFβ1 (Galea et al., 2006)	Pioglitazone, 18 mg/kg/d, po (chow), vs vehicle control, 2 months.	87.81 mg/d	Equal numbers of male and female mice, 2 months old at initiation of treatment.	Pioglitazone inhibited cerebral glucose uptake in control (non-transgenic littermate) mice and failed to reverse TGF1b-mediated inhibition in transgenic mice.
***Diabetes models***				
ICR mice (Jiang et al., 2012) The ICR strain is a general-purpose mouse line. Diabetes was induced by feeding a high fat diet (60% fat, 20% carbohydrate, 20% protein) for 1 month to 10-week-old mice to elicit peripheral insulin resistance and hyperglycemia, followed by injection with streptozotocin (100 mg/kg), to cause insulin deficiency and cerebral hypoinsulinemia.	Pioglitazone, 18 mg/kg/d, po (chow) or 9 mg/kg/d vs vehicle control, for 6 weeks.	87.81 mg/d or 43.9 mg/d	Equal numbers of male and female mice, 16 – 18 weeks old at the initiation of treatment, with similar body weights and degree of hyperglycemia were randomly assigned to equally sized treatment groups. Non-diabetic controls were similarly divided into treatment groups.	HFD/strep-diabetes was associated with memory impairments; pioglitazone treatment improved learning and memory, and reduced soluble Aβ42 and Aβ40, BACE1, NF-κB and RAGE.
Sprague-Dawley rats (Gao et al., 2017) 12-week-old rats were fed 60% fat diet for 20 weeks, then injected with streptozotocin (27 mg/kg).	Pioglitazone, 10 mg/kg/d, po (chow) vs vehicle control, for 10 weeks.	48.78 mg/d	Equal numbers of 20-week-old male and female mice were divided into treatment groups, as described for the ICR mice.	HFD/strep rats exhibited memory impairments versus the control and diabetes+pioglitazone groups, which did not differ from each other. Pioglitazone corrected impaired ERK1/2 mRNA and protein expression caused by hyperglycemia.
***APOE Model***				
*APOE* TR mice (To et al., 2011) 3-month-old male mice fed 60% fat diet for 32 weeks.	Pioglitazone, 20 mg/k/d, po (gavage) vs vehicle control, for 3 weeks.	97.56 mg/d	40-week-old male HF or LF mice were divided into control and pioglitazone treatment groups and dosed for 3 weeks.	HF diet elicited insulin resistance and impaired glucose tolerance; reduced all phospho-tau epitopes in all mice. Neither diet nor pioglitazone affected APP metabolism. In HF mice, pioglitazone was associated with reduced AT8 p-tau in *APOE* ε*3* mice and increased AT8 p-tau in A*POE* ε*4* mice.
***Aging-associated neuropathology***				
Reversal of aging effects in Wistar rats (Cowley et al., 2012)	Rosiglitazone, 3 mg/k/g, po (chow), vs vehicle control, 56 days.	14.63 mg/d	Equal numbers of male and female rats, 22 months old vs 3 months old (control) at the initiation of treatment.	Rosiglitazone improved T1 relaxation times, improved post-synaptic component of LTP, decreased astrogliosis and RANTES expression, mediated by rosiglitazone-enhanced endothelial cell-astrocyte interactions. No effect on microglial activation.
Prevention of aging effects in Wistar rats (Wang et al., 2012)	Rosiglitazone, 6 mg/kg/d, po (chow), vs vehicle control, 40 days.	29.27 mg/d	Equal numbers of male and female rats, 12 – 14 months old (middle aged) vs 1-month-old controls at the initiation of treatment.	Rosiglitazone improved water maze learning, enhanced synaptic plasticity, place cell activity, improved post-synaptic component of LTP, and restored hippocampal GLUT3 expression.
***BOLD imaging Pharmacodynamics***				
Young adult Wistar Rats (Crenshaw et al., 2015)	Pioglitazone, 0.04, 0.08, 0.16, 0.32 mg/kg/d, po (gavage) versus vehicle control, for 2 and 7 days.	0.195, 0.39, 0.78 or 1.56 mg/d	Doses were chosen to bracket the dose used in NCT02284906 (TOMMORROW) (0.8 mg/day), after allometric scaling. Study underpowered for the large number of comparisons.	Resting state functional connectivity increased between two regions after two days of 0.08 mg/kg/day; after seven days 17 connections were changed vs. baseline across all 5 dose groups. On day 7, connectivity between CA1 and ventral thalamus was increased in all pioglitazone doses but was weakest at 0.32 mg/kg/day.

*^*a*^https://www.fda.gov/regulatory-information/search-fda-guidance-documents/estimating-maximum-safe-starting-dose-initial-clinical-trials-therapeutics-adult-healthy-volunteers.*

The first is that the timing and length of treatment are critical variables that that are unique for each disease-related phenotype. The time of treatment in the natural history of the disease can be crucial. Some parameters, such as stimulus-coupled cerebral blood flow and glucose uptake, were normalized in both adult (6 months old at initiation of treatment, before visible signs of plaque pathology or significant deficits in learning and memory) and aged (15 – 18 months old at initiation of treatment) mice, while others, such as reversal learning, were improved in the adult mice, but not in aged mice (Papadopoulos et al., 2013). Other traits, such as spatial learning, were only improved when young mice were treated for extended periods (Badhwar et al., 2013). Second, some parameters respond to short-term treatment, while others do not. Cerebral blood flow was normalized after a short (3 day) drug exposure, but improvements in learning did not occur in that time frame (Badhwar et al., 2013). Third, efficacy becomes more pronounced with time of treatment (Escribano et al., 2010; Chen et al., 2015).

The second lesson is that dose matters. These representative studies employed a wide range of drug concentrations. For pioglitazone, they ranged from 80 to 0.04 mg/kg/day, which, after allometric scaling, represent 390 mg/day, down to 0.8 mg/day for a 60 kg human. For comparison, the recommended starting dose of pioglitazone for treating DM2 is 15 – 30 mg/day. The mode is around 20 mg/kg/day/mouse, equivalent to ∼97mg/day for a 60 kg human. The recommended starting dose for rosiglitazone is 4 mg, but the human equivalent doses used for *in vivo* AD-related studies range from 29 to 58 mg/animal/day. PD/PK-type experiments don’t appear to have been done that would justify the selection of these doses, and, in addition to the dangers of off-target effects, the use of such high doses is problematic because we don’t fully understand the dose-response characteristics of many (if any) of the read-outs. For instance, both human (Knodt et al., 2019) and rat (Crenshaw et al., 2015) studies revealed inverse-U-shaped fMRI BOLD pioglitazone dose response curves. Additionally, Seok et al. found that 2 mg/kg/day pioglitazone was associated with statistically significant improved learning and memory in the Morris water maze test, and reduced IHC-detectable and soluble hippocampal Aβ40 deposits in 9-month-old SAMP8 mice, but the effect on all of these parameters was less at 5 mg/kg/day (Seok et al., 2019). The results of the rat BOLD study also suggest that lower doses were as, if not more, efficacious as higher doses (Crenshaw et al., 2015). These results are reminiscent of cell culture experiments on mitochondrial biogenesis and Aβ clearance (e.g., Miglio et al., 2009; Moon et al., 2012). There is not an agreed-upon explanation for this dose-response pattern, nor can we predict which disease phenotype will respond to PPARγ agonists in this way. Therefore, any evaluation of PPARγ agonists for treating or delaying the onset of AD needs to take into consideration the effect of drug concentration on the parameters being measured.

The lack of PD/PK studies relevant to Alzheimer’s disease, in pre-clinical models or humans, including measurements of target engagement in the brain, contributes to difficulties in planning reliable human studies, and hinders the development of testable theories of drug mechanisms of action on the observed responses. In fact, none of the studies summarized in Table 1 confirmed that, under these treatment regimens, the drugs entered the rodent brain. Rosiglitazone has essentially no BBB penetrance (GSK, unpublished), and pioglitazone has low BBB penetrance (Maeshiba et al., 1997). Yet, pioglitazone ranging from 0.04 to 0.32 mg/kg/d increased functional connectivity between the CA1 region and the ventral thalamus in young adult Wistar rats, but the connectivity fell off at the highest dose (Crenshaw et al., 2015). It appears that either (Longhe, 2020) high pioglitazone concentrations are unnecessary, at least for some responses; or (Patterson, 2018) that some processes are responsive to small amounts that penetrate the BBB and large doses are necessary to overcome the transport barrier (by mass action); or (Zissimopoulos et al., 2015) that some processes respond to actions of the drug outside the BBB. One investigation concluded that rosiglitazone modulates astrocyte behavior *in vivo* indirectly, via regulating interactions between BBB endothelial cells and astrocytes (Cowley et al., 2012). To the best of our knowledge, these observations have not been followed-up. Given the potential for cross-talk between astrocytes and microglia (Clark et al., 2021), such an indirect pathway might also contribute to PPARγ agonist-mediated *in vivo* regulation of microglial function.

In designing *in vivo* efficacy and MOA studies, and to provide guidance for human clinical trials, doses and treatment times should be optimized through implementation of detailed PD/PK studies that include quantification of drug substance and target engagement in the brain and the BBB, as well as in relevant peripheral cells.

## Human Studies

### Observational Cohort Studies

Several longitudinal observational cohort studies have shown that pioglitazone reduces the risk and delays the onset of dementia in the context of type 2 diabetes mellitus. These studies were performed using data extracted from national health insurance records, on subjects who were diagnosed with type 2 diabetes mellitus (DM2) and without dementia (as coded by the International Classification of Diseases, Ninth (ICD-9) or Tenth (ICD-10) editions) on the index date. The index date was the date of first prescription of the drug. In most cases, observations ended after 5 years or when subjects were diagnosed with dementia. Rosiglitazone had a neutral effect (Tseng, 2019). Meta-analysis of these observational studies have been published (Ye et al., 2016; Zhou et al., 2020).

Miller et al. used Department of Veterans Affairs (VA) records to conduct an analysis of US veterans with diabetes but without a recorded diagnosis of AD for two years prior (Miller et al., 2011). Their analysis included subjects prescribed with either rosiglitazone or pioglitazone. The study population was heavily white (79%) and male (98%) type 2 diabetics, who were followed from the time of drug initiation until the first AD diagnosis, which was made using the ICD-9 codes. In this population, the hazard ratio (HR) of thiazolidinedione (TZD) only vs. insulin only was 0.81 (95% CI, 0.73 – 0.89). When insulin and TZD use were combined to improve glycemia control, the HR for insulin followed by TZD was 0.63 (95% CI, 0.53 – 0.74), and for TZD followed by insulin was 0.72 (95% CI, 0.61. – 0.84).

Using German public health insurance company records, Heneka et al. considered only subjects who did not receive insulin and who were dementia-free for two years prior to the index date, which was the date of first pioglitazone use, and they followed subjects for 5 years (Heneka et al., 2015b). The populations they followed were: ≥ 60 years old, free of dementia at the beginning of the study, diabetics not taking pioglitazone, diabetics taking pioglitazone (broken down by length of time on drug), and non-diabetics. Long-term pioglitazone use (receiving pioglitazone prescriptions > 8 quarters) was associated with lower risk for dementia relative to non-diabetics [Relative Risk (RR), 0.53 (95% CI, 0.301 – 0.936, P = 0.029)], while short-term users (<8 quarters) had an RR ∼ nondiabetics (RR, 1.16, P = 0.317). For diabetics without a pioglitazone prescription, the relative risk was 1.23 (P < 0.0001). Neither rosiglitazone nor metformin use altered the risk in this dataset. The RR for insulin use was 1.608 (95% CI, 1.459 – 1.773, P < 0.001).

Chou et al. confirmed that pioglitazone reduced the risk of dementia in patients with DM2, among Taiwanese (Chou et al., 2017). They extracted data from the Longitudinal Health Insurance Database subset of Taiwan’s National Health Insurance Research Database (NHI), for ‘ever pioglitazone’ vs ‘never users’. The cohort were dementia free at the index date, and they incorporated a 5-year follow-up period. The pioglitazone cohort had a higher prevalence of stroke and hypertension than the comparison cohort, which was matched to the pioglitazone cohort by age, sex and index date. They used a ‘defined daily dose’ (DDD, 30 mg/day), as recommended by the World Health Organization, to quantify the daily pioglitazone use. Overall, the risk for dementia was 23% lower in the pioglitazone use group versus in the comparison group; HR = 0.77 (95% CI, 0.62 – 0.95, P = 0.015). The pioglitazone effect was time- and dose-dependent. The hazard ratios were 0.50 (95% CI, 0.34 – 0.75, P = 0.001) in the high cumulative user group (>444 defined daily dose), 0.53 (95% CI, 0.36 – 0.77, P < 0.001) in the long-term user group (>536 days), and 0.66 (95% CI, 0.49 – 0.90, P = 0.009) in the high-mean daily dose user group (>mean daily dose).

Tseng also followed subjects in Taiwan’s NHI, but restricted his analysis to a 2 year follow-up period (Tseng, 2018). Unlike Chou et al., Tseng matched the pioglitazone- and comparison-cohorts for comorbidities, including hypertension, dyslipidemia, ischemic heart disease, peripheral arterial disease, Parkinson’s disease, statin use and other glycemic control agents. Analysis of the unmatched and the matched cohorts confirmed that pioglitazone use was associated with significantly lower risk for dementia. Metformin was also found to be protective in this study, but pioglitazone’s protective effects were independent of metformin. The effect of pioglitazone was largest in patients who had never taken metformin and among those who had taken pioglitazone for > 20 months. In the matched cohort, among patients who had never used metformin, the hazard ratios for ever vs never pioglitazone were: < 11 months: 0.588 (95% CI, 0.272 – 1.273, P = 0.1778), 11 – 19.6 months: 0.690 (95% CI, 0.338 – 1.409, P = 0.3084), > 19.6 months: 0.265 (95% CI, 0.102 – 0.688, P = 0.0064). By contrast, for all patients, the hazard ratio for ever pioglitazone vs never users was 0.716 (95% CI, 0.545 – 0.940, P = 0.163), and the hazard ratios for < 110 months use, 11 – 19.6 months and > 19.6 months use were 0.806 (95% CI, 0.544 – 1.193, P = 0.2809), 0.654 (95% CI, 0.430 – 0.994, P = 0.0467), and 0.694 (95% CI, 0.469 – 1.026, P = 0.067).

Tseng’s findings that metformin was protective are at odds with Heneka et al., who observed no benefits for metformin users. Interestingly, Bohlken et al. also reported that metformin use did not reduce incidence of dementia in a German cohort (Bohlken et al., 2018). Their study relied on the German Disease Analyzer database (IQVIA), representative of General Practices (as opposed to all sources of health case, including Neurology), and involved a disease cohort that was dementia-free for at least one year prior to the index date. The ‘ever’ and ‘never’ drug cohorts were matched for age, sex and comorbidities. The odds ratio of developing dementia for those taking glitazones (pioglitazone or rosiglitazone) was 0.80 (95% CI, 0.68 – 0.95, P = 0.011) and the odds ratio for metformin was 0.96 (95% CI, 0.88 – 1.04, P = 0.153). The discordance between the German and Taiwanese metformin results could reflect differences in dementia subtypes (Neff et al., 2021), other genetic background differences of the ethnic groups represented in the respective databases, comorbidities of the ‘ever’ and ‘never’ groups in the respective studies, the severity of diabetes, or methodological differences in data collection and analysis.

Numerous observational studies of other hypoglycemic agents, including insulin, glycosidase inhibitors, metformin, sulfonylureas and DPP-4 inhibitors have been conducted, and the results have been examined and summarized in two meta-analysis (Ye et al., 2016; Zhou et al., 2020). Generally, the DPP-4 inhibitors were associated with the lowest risk of dementia, followed by metformin and the thiazolidinediones, which lumped pioglitazone and rosiglitazone together (Zhou et al., 2020). Another study has shown that no hypoglycemic agents, including thiazolidinediones or metformin, delayed the onset of AD (Imfeld et al., 2012). Because the effects of rosiglitazone are neutral (Tseng, 2019), lumping it together with pioglitazone produces confounding results in these meta-analyses. DPP-4 inhibitors block the degradation of the incretin hormones (GIP, GLP-1), which regulate microglial function (Spielman et al., 2017). Because incretins trigger PPARγ expression (Svegliati-Baroni et al., 2011; Onuma et al., 2014), it is not possible to disentangle the PPARγ and incretin contributions to the DPP-4 effects evident in these data.

Overall, the longitudinal cohort studies demonstrate pioglitazone use is associated with a reduced risk for dementia in populations of adult-onset diabetics, and the effect is time and dose dependent. These results are in line with the pilot clinical studies conducted using DM2 cases (Hanyu et al., 2009, 2010; Sato et al., 2011). The strength of the ‘never vs ever’ longitudinal cohort studies is they approximated placebo controlled, clinical trials that matched subjects for age, sex and co-morbidities and had realistic follow-up periods (2 – 5 years). However, they all relied on ICD diagnostic codes to define dementia and often there was a lag between onset of dementia and diagnosis. Heneka et al. attempted to overcome that shortcoming by adopting a multi-layer approach to diagnosis. Additionally, none of these studies accounted for *APOE* or other genetic risk factors, life-style factors, or the severity of diabetes and the degree of insulin resistance and glycemic control. To overcome the difficulties of these observational studies and of the small-scale pilot clinical studies with DM2 (section 10.2), a large-scale blinded, placebo controlled clinical trial in populations with adult-onset diabetes, that are at increased risk of developing Alzheimer’s disease, seems warranted.

### Clinical Studies

Table 2 summarizes twelve clinical trials that reported data evaluating thiazolidinedione PPARγ agonists as therapeutics for Alzheimer’s disease. We will focus on those studies that highlight important points. We include two pharmacodynamics studies using measures of brain energetics as readouts, two small studies that included measures of drug effects on Aβ peptide levels, two that correlated drug effects on insulin-lowering, and three small studies evaluating the efficacy of pioglitazone in volunteers with pre-existing DM2; we did not include in the table an additional study reporting similar outcomes by this group, but we do discuss it below. Aside from the studies in DM2 patients, all of the studies excluded individuals with a history of diabetes or who were taking medications to control glucose. Two of the studies were prevention trials with participants who were free of dementia at the time of enrollment, that reflected one of the important lessons from the pre-clinical studies, that beginning treatment before the onset of AD-related pathology preserved learning and memory (Badhwar et al., 2013). The rest were treatment studies that involved subjects with mild-to-moderate Alzheimer’s disease or mild cognitive impairment at the outset. With the exception of NCT00348309, NCT00348140 and NCT01931566, all of the studies may have been underpowered or conducted for too short a duration relative to the conversion rate in the controls from normal cognition to MCI or mild-AD.

**TABLE 2:** Summary of PPARγ agonist clinical trial results for Alzheimer’s disease.

**Study**	**Treatment**	**Study Design**	**Population**	**Results**
**A. Rosiglitazone**				
**A.1. Phase 2 Intervention**				
Preserved cognition in patients with early Alzheimer disease and amnestic mild cognitive impairment during treatment with rosiglitazone (Watson et al., 2005)	Rosiglitazone 4 mg daily, vs placebo.	24-week, placebo-controlled, double blind, parallel-group study in subjects with early AD and Amnestic MCI. Outcome measures: cognition, plasma insulin, plasma Aβ.	Placebo, N = 10, rosiglitazone, N = 20. Average age, 73 years, 70% F, 100% White. Baseline insulin, 8.1 μU/mL; MMSE mean baseline, 23. Subjects taking medications to control glucose were excluded.	Rosiglitazone was statistically associated with better delayed recall at 4 and 6 months, selective attention at 6 months, stable plasma Aβ42, Aβ40, and Aβ42/Aβ40 ratio.
Efficacy of rosiglitazone in a genetically defined population with mild-to-moderate Alzheimer’s disease (Risner et al., 2006)	Rosiglitazone 2, 4 or 8 mg daily vs placebo.	24-week, placebo-controlled, double blind, parallel-group pilot study in subjects with mild-to-moderate AD (phase 2). Outcome measures: ADAS-Cog, CIBIC+	Average N = 128, average age, 70.7 years, 60% F, 100% White. Balanced for *APOE* ε4. Baseline insulin, 14.2 μU/mL. MMSE mean baseline, 21.3. Subjects with history of T1DM or T2DM, or with fasting glucose ≥ 7mM or HbA1c ≥ 8.5% were excluded.	*Overall*: no statistically significant effect of rosiglitazone on outcome measures. *In APOE ε4 non-carriers*, treatment with 8 mg rosiglitazone was statistically associated with improved ADAS-Cog.
*NCT00265148* Effects of rosiglitazone on cognition and cerebral glucose utilization in subjects with mild to moderate Alzheimer’s disease (Tzimopoulou et al., 2010)	Rosiglitazone 4 mg daily for one month, increasing to 8 mg daily for the remainder of the study, vs placebo.	52-week parallel group, double blind, phase 2 study. Outcome measures: 12-month cerebral glucose metabolic rate change, brain volume, ADAS-Cog, CIBIC+	Average N = 31 completed the study, average age, 71.25 years, 46.2% F, 94.9% White. Balanced for *APOE* ε4. Subjects with history of T1DM or T2DM or taking medications* to control glucose were excluded.	No sustained treatment effect on total or regional glucose metabolic rate or brain volume; no effect on ADAS-Cog or CIBIC+.
**A.2. Phase 2 – Diabetes**				
Rosiglitazone and cognitive stability in older individuals with type 2 diabetes and MCI (Abbatecola et al., 2010)	Rosiglitazone 4 mg daily, vs metformin, 500 mg daily, vs. rosiglitazone + metformin vs. diet.	36-week, prospective, randomized, open-controlled study in subjects with mild-to-moderate AD in association with T2DM. Outcome measures: changes in neuropsychological test scores and metabolic control parameters (FIRI, FPG, HbA1C).	Average N = 32.2, average age, 76 years, 45% F; FPG mean baseline, 8.44 mmol/L; FIRI, 148 pmol/L; mean baseline HbA1C, 7.5%; MMSE mean baseline, 24; TMT-A mean baseline, 67.6; TMT-B mean baseline, 161.1; DIFFBA mean base line, 101.2; RAVLT mean baseline, 24.5.	Metformin/rosiglitazone combination stabilized all neuropsychological tests. Metformin stabilized MMSE, TMT-A, TMT-B; diet stabilized MMSE, TMT-A. In linear-fixed effects model, FIRI x time correlated with metformin/rosiglitazone RAVLT.
**A.3. Phase 3 Intervention**				
*NCT00428090* Rosiglitazone (Extended Release Tablets) as monotherapy in subjects with mild to moderate Alzheimer’s disease (Gold et al., 2010)	Rosiglitazone 2 or 8 mg extended release, daily vs placebo (REFLECT-1).	24-week, double blind, double dummy, randomized, parallel group phase 3 study, stratified for APOE ε4 status in subjects with mild-to-moderate AD. Outcome measures: ADAS-Cog, CIBIC+	Average N = 159, average age, 72.3 years, 37% F, 72% White, balanced for *APOE* ε4. ADAS-Cog mean baseline, 19.1. Subjects with history of T1DM or T2DM or taking medications* to control glucose were excluded.	No statistically or clinically significant effect of either rosiglitazone dose in full population; no significant evidence of interaction between treatment with rosiglitazone and APOE genotype.
*NCT00348309/NCT00348140* Rosiglitazone (Extended Release Tablets) As Adjunctive Therapy For Subjects With Mild To Moderate Alzheimer’s Disease (Harrington et al., 2011)	Rosiglitazone 2 or 8 mg extended release, as adjunctive to donepezil (REFLECT-2), or adjunctive to any AChEIs (REFLECT-3).	48-week, double blind, randomized, placebo-controlled, parallel group, phase 3 studies, stratified for APOE ε4 status in subjects with mild-to-moderate probable AD. Outcome measures: ADAS-Cog, CDR-SB.	*NCT00348309* (REFLECT-2): Average N = 464, average age, 74.1 years, 60% F, 90.67% White, balanced for *APOE* ε4. ADAS-Cog mean baseline, 25.3; MMSE mean baseline, 19.46. *NCT00348140* (REFLECT-3): Average N = 476.3, average, 73.9 years old, 55.6% F, 91.76% White, balanced for *APOE* ε4. ADAS-Cog mean baseline, 24.1; MMSE mean baseline, 19.7. Subjects with history of T1DM or subjects with T2DM taking medications* to control glucose were excluded.	No statistically or clinically significant effect of either rosiglitazone dose in full population; no significant evidence of interaction between treatment with rosiglitazone and APOE genotype in either REFLECT-2 or REFLECT-3.
**B. Pioglitazone**				
**B.1. Phase 1 - Dose-ranging**				
*NCT01456117* Study to assess the effects of daily administration of pioglitazone on brain hemodynamics in cognitively healthy elderly subjects (Knodt et al., 2019)	Pioglitazone, 0.6 mg, 2.1 mg, 3.9 mg, 6.0 mg daily, vs. placebo	A 2-week, multiple-dose, single-blind, randomized, parallel design, placebo-controlled, phase 1 dose-ranging study. Outcome measure: episodic memory-related hippocampal activity, measured via blood oxygen level-dependent (BOLD) functional magnetic resonance imaging.	Average N = 11, average age = 66.08 years, 71% F, 85.45% White. CERAD-WLM mean baseline, 8.02, TMT-B mean baseline, 97.58. Diabetic subjects taking medications to control blood glucose*, or with HbA1C > 6% were excluded.	Statistical association of 0.6 mg/day pioglitazone with increased right hippocampal activation during encoding of novel face-name pairs at day 7 and day 14, relative to baseline. No statistically significant improvement at 2.1, 3.9 or 6.0 mg/day.
**B.2. Pilot - Diabetes**				
Role of tumor necrosis factor-alpha in cognitive improvement after peroxisome proliferator activator receptor gamma agonist pioglitazone treatment in Alzheimer’s disease (Hanyu et al., 2010)	Pioglitazone, 15 mg daily vs. none.	24-week, prospective, randomized, open-controlled study, in subjects with mild-to-moderate Alzheimer’s disease in association with T2DM. Outcome measures: ADAS-JCog, MMSE, TNFα, IL-6, C-reactive protein.	N = 17 for both groups, average age 78.7 years, 50% F, 100% White, balanced for APOE ε4 and donepezil use; MMSE mean baseline, 21.85; ADAS-JCog mean baseline, 15.65; TNFα mean baseline, 1.38 pg/mL; IL-6 mean baseline, 2.62 pg/mL; C-reactive protein mean baseline, 0.08 mg/dL.	Pioglitazone was statistically associated with improved ADAS-JCog and TNFα, and changes in ADAS-JCog were correlated with changes in TNFα.
Efficacy of PPARγ agonist pioglitazone in mild Alzheimer disease (Sato et al., 2011)	Pioglitazone, 15 or 30 mg daily vs. none.	24-week prospective randomized, open-controlled study in subjects with mild-to-moderate Alzheimer disease in association with T2DM. Outcome measures: ADAS-JCog, MMSE, WMS-R, rCBF, plasma Aβ40 and Aβ42, HOMA-R, HbA1c, FIRI.	N = 21 for both groups, average age, 77.5 years, 52% F, 100% White, balanced for *APOE* ε4; balanced for other hypoglycemic agents, donepezil.	Pioglitazone was statistically associated with improved MMSE, ADAS-JCog and WMS-R, with improved blood flow in the parietal lobe, and with improved metabolic factors. The plasma Aβ40/Aβ42 ratio did not change in the pioglitazone group and increased in the control group. ADAS-JCog significantly worsened in the control group.
**B.3. Phase 2 Intervention**				
*NCT00982202* Pioglitazone in Alzheimer’s disease safety trial (Geldmacher et al., 2011)	Pioglitazone, 15 mg daily, escalating weekly to 45 mg daily, vs. placebo.	72-week, double-blind, randomized, placebo-controlled, group comparison study of mild-to-moderate probable Alzheimer’s disease Outcome measures (collected at 3-month intervals): (Longhe, 2020). Measures of cognition, including ADAS-Cog, CDR-SB. (Patterson, 2018). Estimate for effect size calculations.	Average N = 14.5, average age, 70.95 years, 62% F; MMSE mean baseline, 21; ADAS-Cog mean baseline, 21; CDR-SB mean baseline, 5.8.	Pioglitazone was not statistically associated with any improved measure of cognition; the adjusted mean for ADAS-Cog per month was lower in the pioglitazone group, but not statistically significant. For α = 0.05 and power = 0.80, sample sizes of 340 (170 pio, 170 placebo) and 155 (78 pio, 77 placebo) subjects would be required for their estimated regression coefficients of the pioglitazone effect on ADAS-Cog (-0.746) and CDR-SB (-0.354), respectively, to be significant.
*NCT00736996* Pioglitazone and exercise effects on older adults with MCI and metabolic syndrome (POEM) (Hildreth et al., 2015)	Pioglitazone, 15 mg daily escalating to 45 mg daily after one month, versus placebo; or 45-75 minutes exercise training 3X/week, vs. *status quo* exercise. Exercise regimen initiated at 50-60% HR max, escalated to 80-85% HR max over the course of the study.	24-week double-blind, randomized, placebo-controlled pilot study in sedentary adults with MCI and central obesity. Outcome measures: Change in baseline for cognition, insulin clamp, body composition, metabolic and inflammatory markers.	Average N = 22, average age 65.6 years, 51.8% F, 87.8% White, balanced for *APOE* ε4; average compliance, pioglitazone, 76%, placebo, 89%; glucose mean baseline, 101 mg/dL; insulin mean baseline, 16.1 μU/mL; C-reactive protein mean baseline, 3 mg/L; IL-6 mean baseline, 1.7 pg/mL; TNFα mean baseline, 1.56 pg/mL; MMSE mean baseline, 28.6; ADAS-Cog mean baseline, 6.	Pioglitazone was not statistically associated with any improved measure of cognition; performance on the Visual Reproduction Test; scores worsened in the pioglitazone group vs. placebo; ADAS-Cog improved with exercise (-1.3 EX vs. -0.3 CON; P = 0.05). No statistically significant correlations between glucose disposal rates and cognitive performance.
**B.4. Phase 3 Prevention**				
*NCT01931566* A study to simultaneously qualify a biomarker algorithm for prognosis of risk of developing MCI Due to AD and to test the safety and efficacy of pioglitazone to delay the onset of MCI due to AD in cognitively normal subjects (Alexander et al., 2019; Burns et al., 2019) *NOTE: The full publication describing this study was under review when the current paper was submitted.*	Pioglitazone, 0.8 mg extended release daily, vs placebo.	Event-driven (anticipated 5 yr.), double-blind, randomized, parallel group placebo-controlled Phase 3 prevention study in cognitively normal adults susceptible for AD (*APOE, TOMM40* genotypes and age). Outcome measures: Delay onset of MCI in normal participants who are at increased risk due to age and genetic risk factors.	N = 433 low-risk placebo, 1516 high-risk placebo, 1545 high risk pioglitazone. Average age, 73.1 years, 56.16% F, 96.6% White; average *APOE* ε*4* carriage in the high-risk groups, 92.45%; MMSE mean baseline, 28.56. Outcome measure: Time to diagnosis of MCI due to AD for pioglitazone-treated subjects vs placebo in high-risk stratum. Pre-specified futility threshold, 30% conditional probability that a 40% treatment difference would be detected.	Study terminated due to futility analysis. After 1278 days, total events in placebo, 46; total events with pioglitazone, 39. Pioglitazone risk ratio vs placebo was 0.8 (95% CI, 0.45 – 1.4), P = 0.307; post-hoc subgroup analysis suggests possible benefit of pioglitazone for males.

ADAS-Cog, Alzheimer’s Disease Assessment Scale-Cognitive Subscale; ADAS-JCog, Japanese version of the ADAS-Cog; ATP III, Adult treatment panel III criteria for central obesity; rCBF, regional cerebral blood flow; CDR-SB, Clinical Dementia Rating scale-Sum of Boxes; CIBIC+, Clinician’s interview-based impression of change with caregiver input; DIFFBA, TMT-A minus TMT-B – a measure of cognitive efficiency; FIRI, fasting immunoreactive insulin; FPG, fasting plasma glucose; HOMA-R, homeostatic model assessment for insulin resistance; MCI, mild cognitive impairment; MMSE, Mini-mental state examination; RAVLT, Rey Auditory-verbal learning test; T1DM, type 1 diabetes mellitus; T2DM, type 2 diabetes mellitus; TNFα, tumor necrosis factor-alpha; TMT-A, TMT-B, Trail marking test A and Trail marking test-B, respectively. *Insulin, sulfonylureas, PPARγ agonists or glitinides.

Several small pilot studies evaluated the efficacy of rosiglitazone and pioglitazone in the context of the metabolic risk factors insulin resistance and type 2 diabetes mellitus (Luchsinger et al., 2004; Biessels et al., 2006; Muller et al., 2007; Xu et al., 2009; Willette et al., 2015a, b; Wu et al., 2016). Watson et al. enrolled volunteers with early AD and amnestic MCI for a 24-week trial with rosiglitazone (Watson et al., 2005). These volunteers were also mildly insulin resistant, based on the HOMA-IR scores calculated from the reported average baseline insulin and glucose values (calculated 2.0; scores > 1.9 are mildly insulin resistant) (Matthews et al., 1985). In addition to changes from baseline cognitive scores, these authors they quantified plasma insulin and, unlike most of the other studies that we located, the plasma biomarkers Aβ42 and Aβ40. Over the course of the 6-month trial, rosiglitazone preserved delayed memory scores and selective attention, while memory deteriorated in the control group (Watson et al., 2005). The Aβ42/Aβ40 ratio fell in the placebo group but was stabilized by rosiglitazone. Rosiglitazone also elicited a small but statistically significant drop in peripheral insulin, and the degree of memory preservation and error rates on the interference test were inversely related to changes in plasma insulin levels. Risner et al. also found suggestive evidence of an interaction between changes in insulin levels and cognition (Risner et al., 2006). This effect may be related to the correction of central hypoinsulinemia, which peripheral hyperinsulinemia causes (Baura et al., 1996). Glucose levels rose, but the change did not attain statistical significance.

*APOE* ε*4* is the most significant genetic risk factor for developing late-onset Alzheimer’s disease, and several studies investigated whether there was an interaction of *APOE* ε*4* carriage with rosiglitazone or pioglitazone effects on cognition. Risner et al. conduced a small, 24-week, dose-response study (average N = 128) (Risner et al., 2006). Overall, rosiglitazone did not have a significant effect on cognition, but tests for interaction between ADAS-Cog score and APOE status were significant. Cognition improved at the highest rosiglitazone dose (8 mg) in *APOE* ε*4* negative subjects. Notably, *APOE* ε*4* negative subjects also experienced a greater drop in plasma insulin elicited by 8 mg rosiglitazone than subjects who carried at least one *APOE ε*4 allele. The relationship between insulin lowering and cognitive improvement is reminiscent of Watson et al. (2005), but unlike in the latter study, the interaction was not formally analyzed here.

GlaxoSmithKline studied the interaction between *APOE* status and rosiglitazone efficacy further in two larger-scale, dose-response trials, which compared the efficacy of low (2 mg) versus high (8 mg) rosiglitazone in test populations that were stratified by APOE status, as adjunctive therapy to AChEIs (Gold et al., 2010; Harrington et al., 2011). NCT00428090 ran for 24 weeks, with average N = 124 subjects (Gold et al., 2010), and the NCT00348309 and NCT00348140 studies ran for 48 weeks with average N = 464 subjects (Harrington et al., 2011). Rosiglitazone did not have a statistically significant effect on cognition in either *APOE* ε*4*+ or *APOE* ε*4*- subjects at either dose, in either trial. There was essentially no difference in HbA1c values between the two rosiglitazone concentrations in Gold et al. (2010) and increased with increasing dose of rosiglitazone in Harrington et al. (2011). The authors did not report any statistical interaction between these changes and scores for cognition. Fasting glucose and fasting insulin values were not measured in any of these studies.

As demonstrated in volunteers with DM2 (Sato et al., 2011) as well as in mouse models of AD (Nicolakakis et al., 2008; Papadopoulos et al., 2013), PPARγ agonists promote central glucose metabolism. Tzimopoulou et al. measured cerebral glucose metabolic rates (CMRglu) and brain atrophy as pharmacodynamics markers, of central rosiglitazone action. The volunteers for this study had mild-to-moderate Alzheimer’s disease, and were age- and sex-matched with cognitively normal controls for a 52-week trial (Tzimopoulou et al., 2010). Rosiglitazone (8 mg extended release/day) was statistically associated with a modest (1.5%) increase in glucose utilization, compared with a 4.7% decrease for the placebo over the first month of treatment. However, this immediate increase was not robust and the mean CMRglu rates decreased in both groups over the remaining 11 months of the trial. Although the rate of decline of CMRglu was lower in the rosiglitazone group than in the placebo, the trend was only suggestive, and there was no evidence that rosiglitazone affected changes in brain volume or cognition. *APOE* ε*4* carriage did not affect any outcome. As above for the REFLECT studies, no fasting insulin or glucose values were recorded, so it was not possible to assess interaction between changes in insulin level or insulin resistance and cognition or these pharmacodynamics markers.

In a series of small pilot studies with DM2 cases, Hanyu’s group reported that pioglitazone (15 – 30 mg/day) improved cognitive measures in type 2 diabetics after 6 months; ADAS-JCog scores improved in DM2 cases taking the drug, but worsened in the control diabetics who did not (Hanyu et al., 2009, 2010; Sato et al., 2011). Regional cerebral blood flow also improved with pioglitazone (Sato et al., 2011), as did peripheral TNFα levels (Hanyu et al., 2010). Pioglitazone stabilized the Ab42/Ab40 ratio, which decreased in the controls (Sato et al., 2011).

While Hanyu’s group monitored cases with pre-existing DM2 to evaluate pioglitazone’s effectiveness in delaying cognitive decline, Abbatecola et al. took a different tack, to learn if a PPARγ agonist could ameliorate pre-existing MCI. Another difference was they studied the combination therapy of rosiglitazone added to metformin, vs monotherapy of metformin alone or diet alone to control glycemia. Combining rosiglitazone with metformin was superior to metformin alone and diet alone in slowing cognitive decline (Abbatecola et al., 2010). These data are consistent with the longitudinal cohort study, showing a trend toward increased protection in metformin users in the first 12 months following the addition of rosiglitazone (Tseng, 2019).

Of the remaining three studies, one was a pharmacodynamics study and two were prevention studies. Hildreth et al. directly investigated the role of a metabolic risk factor, insulin resistance, on cognitive impairment in genetically low-risk populations, using a high pharmacological dose of pioglitazone. The TOMMORROW study measured (NCT01931566) the efficacy of pioglitazone to delay the onset of cognitive impairment in a population that was metabolically robust but genetically at risk for developing late-onset Alzheimer’s disease; it involved a very low pioglitazone dose established with the help of the PD study.

Knodt et al. used BOLD fMRI as a pharmacodynamics tool, to determine pioglitazone’s effect on the hippocampal function and as a tool to inform dose selection for the TOMMORROW study (NCT01931566) (Knodt et al., 2019). Healthy, cognitively normal volunteers received daily for 14 days vehicle (placebo), or 0.6 mg, 2.1 mg, 3.9 mg or 6.0 mg pioglitazone. For perspective, the starting dose, and smallest tablet size, for treating DM2 is 15 mg, and these doses ranged from 0.4 to 40% of that dose. Overall, reaction times for correctly recalled face-name pairs were negatively correlated with activity in both the right and left hippocampus during encoding. 0.6 mg pioglitazone was associated with increased right hippocampal activation from baseline to day 7 and from baseline to day 14. The placebo group exhibited decreased right hippocampal activation from day 7 to day 14. These data support that pioglitazone has an effect in conscious humans on brain function, and moreover suggest a hormetic dose-response effect on hippocampal function, similar to what was observed in the rat BOLD study for interactions between the CA1 region and hypothalamus and ventral thalamus (Crenshaw et al., 2015). Together with the findings from Hanyu’s group summarized above and the observational cohort studies, that involved clinical levels of pioglitazone (Heneka et al., 2015b; Chou et al., 2017; Tseng, 2018), these results suggest the overall salutary effect of pioglitazone on risk of dementia may be mediated through multiple targets, each responsive to a unique pioglitazone concentration range.

The POEM study evaluated and compared the efficacies of exercise and pioglitazone on changes in cognition scores and on metabolic parameters (Hildreth et al., 2015). Additionally, they measured circulating markers of inflammation (CRP, IL-6 and TNFα). As in Watson et al. (2005), the participants in this study were insulin resistant at baseline (HOMA-IR score, 4.0), but unlike Watson et al. they were cognitively normal (mean baseline MMSE, 28.6). Neither pioglitazone nor exercise affected circulating inflammatory markers. Fasting insulin and insulin resistance, as measured by euglycemic-hyperinsulinemic clamp, improved in the pioglitazone group, but neither exercise nor pioglitazone affected cognitive performance, and there was no interaction between improved glucose disposal rate and any domain of cognitive performance. Cognitive performance did improve in *APOE* ε*4*-negative participants, but the change was not statistically significant. As Hildreth et al. pointed out, if there was any cognitive impairment among the participants at baseline, it was very mild. With only an average of 22 subjects, followed for only 6 months, likely there would have been too few conversions from normal cognition to MCI in the placebo group to detect possible effects of pioglitazone.

The TOMMOROW study (NCT01931566) was designed to determine if low dose (0.8 mg/day, extended release) pioglitazone would delay-of-onset of mild clinical impairment due to Alzheimer’s disease, in cognitively and metabolically normal subjects who, due to genetic risk factors, are at high risk of developing MDI due to AD within 5 years. It involved 3500 participants; aged 65 – 83. Subjects were assigned as either low or high risk to develop MCI due to AD in the subsequent 5 years, stratifying risk by age at entry and genotypes at *APOE* and *TOMM40 ‘523* loci. High-risk subjects, most of whom carried at least one *APOE* ε*4* allele, were assigned to either the placebo or treatment arm (average N = 1530). The baseline average MMSE score was 28.56, and volunteers with a history of diabetes or who were taking drugs that affected glycemia were excluded. The study outcome was the delay of onset of MCI, and it was sufficiently powered to detect a 30% difference in change from base line over the anticipated running time of 5 years had there been one. However, after the study was initiated the futility criteria was changed to 40% difference, and futility analysis led to an early termination of the trial, when the majority of subject had less than 3 years’ drug exposure. Pioglitazone in high-risk non-Hispanic/Latino Caucasian subjects did not have a statistically significant effect different from placebo (39/1430 [2⋅7%] *vs* 46/1406 [3⋅3%]; HR 0⋅80; 99% CI, 0⋅45 – 1⋅40; p = 0⋅307). Although not statistically significant, a pre-specified sex subgroup analysis revealed potential differences in male subjects (pioglitazone HR 0⋅56; 95% CI, 0⋅30–1⋅06; p = 0⋅074) (Alexander et al., 2019). No pharmacodynamic measures were collected, including plasma Aβ peptide levels. No metabolic parameters aside from HbA1c were collected, and analysis of possible interactions between changes in performance on the cognitive battery and changes in fasting insulin or insulin resistance is not possible.

In addition to the clinical studies summarized in Table 2 for PPARγ agonists, a dual PPARδ/PPARγ agonist currently is being evaluated for its effects on the risk of developing dementia in subjects with mild to moderate AD. T3D-959 is 15X more potent against PPARδ than PPARγ. PPARδ agonists are hypothesized to reduce risk for AD because they regulate glucose and fatty acid utilization and enhance anti-oxidant and anti-inflammatory signaling (Liu Y. et al., 2018). In an exploratory phase IIb study, T3D-959 increased cerebral glucose utilization, and provided suggestive improvement in cognitive assessments (Chamberlain et al., 2020). A double-blind placebo-controlled phase 2 dose-ranging study is currently underway. Its primary outcomes are effects on cognition and global function, and exploratory measures include plasma Aβ 42/40 ratio, Nfl and tau, and cerebral glucose utilization (Clinicaltrials.gov NCT04251182) (Didsbury et al., 2020).

Overall, pioglitazone and rosiglitazone were ineffective in restoring cognitive function or in delaying the onset of MCI due to Alzheimer’s disease in non-DM2 volunteers. However, several caveats should be considered before we can consider this a settled issue. First, most of these studies were insufficiently powered or were not conducted long enough to detect changes with statistical confidence (Watson et al., 2005; Hanyu et al., 2009, 2010; Abbatecola et al., 2010; Tzimopoulou et al., 2010; Geldmacher et al., 2011; Sato et al., 2011; Hildreth et al., 2015; Knodt et al., 2019). For instance, Geldmacher et al. conducted an 18-month, Phase 2 pioglitazone (15 mg/day initially, escalating to 45 mg/day after one month) safety study in volunteers with Alzheimer’s disease, that also permitted effect-size calculations (Geldmacher et al., 2011). They enrolled an average of 14.5 volunteers in each cohort, with average MMSE baseline scores of 21. They administered five separate cognitive tests every 6 months, and calculated the regression coefficients for a multilevel model for each test. The ADAS-Cog parameter they obtained (−0.746) was not significant, nor were the ones for any other of the tests they ran. From their data, Geldmacher et al. estimated the average cohort size would have to be ∼170 for the observed ADAS-Cog parameter to have been significant, with α = 0.05 and power = 0.8. We cannot directly compare this study with any of the others, it begs the question of what the results would be if similar calculations were applied to the other studies.

Second, both Watson et al. and Risner et al. showed that rosiglitazone had a positive effect on cognitive decline after taking changes in fasting insulin into account, and Risner et al. also found that changes in fasting insulin were more extensive in individuals without an *APOE* ε*4* allele. Peripheral hyperinsulinemia causes central hypoinsulinemia (Baura et al., 1996), which underlies this effect. Several groups (Watson et al., 2005; Gold et al., 2010; Tzimopoulou et al., 2010; Harrington et al., 2011) measured fasting glucose and/or HbA1c, but these measurements were not useful. Insulin itself is a better covariate than glucose or its surrogates for use in clinical trials of PPARγ action. Yet most of the studies subsequent to Risner et al. (2006) failed to take drug effects on fasting insulin into account.

Third, the concordance of rosiglitazone’s effects on cognition and the Aβ42/Aβ40 ratio in Watson et al. (2005) and Sato et al. (2011) suggests that Aβ peptides are useful biomarkers for monitoring PPARγ - mediated effects in clinical trials of this sort, especially since the mechanism underlying the PPARγ effect on APP processing and Aβ release is well-understood, and standardized blood tests are available for clinical use. PPARγ agonists also inhibit tau phosphorylation, and because plasma p-tau 181 is associated with the metabolic and cognitive deficits associated with AD (Lussier et al., 2021), incorporation of a standardized, sensitive test for p-tau 181 (Karikari et al., 2020) would also be useful. The attempt to use cerebral glucose metabolism as a pharmacodynamics marker for PPARγ agonist efficacy in AD (Tzimopoulou et al., 2010) was unsuccessful due to study limitations. While Tzimopoulou et al. revealed a sustained protective effect of rosiglitazone on glucose metabolism (Tzimopoulou et al., 2010), the study did not run long enough for statistically or clinically meaningful conclusions to be drawn. BOLD fMRI as a PD marker also has theoretical justifications summarized in Knodt et al. (2019), but only one pre-clinical study has been published related to pioglitazone’s effects on BOLD signaling (Crenshaw et al., 2015), and its suitability vis a vis more established biomarkers has not been established. In light of the difficulties with these PD biomarkers, and the informativeness of alternations in Aβ levels, it is disappointing that plasma Aβ42 and Aβ40 measurements were not incorporated more widely in clinical trials testing the efficacy of PPARγ agonists to delay the onset of dementia due to AD.

It would be informative to both our understanding of the underlying pathophysiology of Alzheimer’s disease and for drug development purposes to learn if the relationships between changes in cognitive scores and changed fasting plasma insulin, and changed Aβ42 and Aβ40 peptide levels recorded in the Pilot and Phase 2 trials, were simply type I errors. As we’ve shown in this review, the relationship between each of these parameters and PPARγ MOA is empirically justified, and we recommend that future large-scale AD drug trials of PPARγ agonists or of the PPARγ/δ dual agonist T3D-959, incorporate measurements of fasting insulin, Aβ peptides and p-tau 181 as covariates.

## Conclusion

Pioglitazone represents ‘polypharmacy in a pill’, and improves multiple etiopathologic determinants of Alzheimer’s disease, including inflammation and oxidative stress, microglial defects, the development of amyloid plaques and neurofibrillary tangles, impaired cerebral glucose consumption and mitochondrial dysfunction, involving suppressed bioenergetics and disrupted dynamics. Pre-clinical studies have shown pioglitazone improves learning and memory, which correlate with improved synaptic activity and reduced amyloid and tau pathology, and better effects are seen when treatment is initiated before the onset of AD pathology. Longitudinal cohort studies have shown that pioglitazone is a time- and dose-dependent protective factor in individuals with DM2. These results are consistent with small scale, pilot studies in DM2 cases that showed pioglitazone increased cerebral blood flow as well as delayed the onset of dementia. Most of the clinical studies that have been conducted to date have been small and underpowered, or have not run long enough to be decisive. However, they are suggestive that pioglitazone’s effects on cognition interacts with its effects on insulin lowering, even in cases without DM2.

## Author Contributions

WG wrote the manuscript. All authors discussed and edited the manuscript, and read and approved the final manuscript.

## Conflict of Interest

AS is President and CEO of Zinfandel Pharmaceuticals, Inc. DB is Senior Vice President and COO of Zinfandel Pharmaceuticals, Inc. WG has received consulting fees from Zinfandel Pharmaceuticals, Inc.
